# Plasticity versus specificity in RTK signalling modalities for distinct biological outcomes in motor neurons

**DOI:** 10.1186/s12915-014-0056-6

**Published:** 2014-08-14

**Authors:** Nathalie Caruso, Balazs Herberth, Fabienne Lamballe, Vilma Arce-Gorvel, Flavio Maina, Françoise Helmbacher

**Affiliations:** Aix-Marseille Université, IBDM, CNRS UMR 7288, Parc Scientifique de Luminy, Case 907, 13288 Marseille, France

**Keywords:** Motor neurons, RTK signalling, Survival, Cell fate specification, Development, HGF/Met

## Abstract

**Background:**

Multiple growth factors are known to control several aspects of neuronal biology, consecutively acting as morphogens to diversify neuronal fates, as guidance cues for axonal growth, and as modulators of survival or death to regulate neuronal numbers. The multiplicity of neuronal types is permitted by the combinatorial usage of growth factor receptors, each of which is expressed in distinct and overlapping subsets of neurons, and by the multitasking role of growth factor receptors, which recruit multiple signalling cascades differentially required for distinct biological outcomes. We have explored signalling robustness in cells where a given receptor tyrosine kinase (RTK) elicits qualitatively distinct outcomes. As the HGF/Met system regulates several biological responses in motor neurons (MN) during neuromuscular development, we have investigated the signalling modalities through which the HGF/Met system impacts on MN biology, and the degree of robustness of each of these functions, when challenged with substitutions of signalling pathways.

**Results:**

Using a set of mouse lines carrying signalling mutations that change the Met phosphotyrosine binding preferences, we have asked whether distinct functions of Met in several MN subtypes require specific signalling pathways, and to which extent signalling plasticity allows a pleiotropic system to exert distinct developmental outcomes. The differential ability of signalling mutants to promote muscle migration versus axonal growth allowed us to uncouple an indirect effect of HGF/Met signalling on nerve growth through the regulation of muscle size from a direct regulation of motor growth via the PI3 kinase (PI3K), but not Src kinase, pathway. Furthermore, we found that HGF/Met-triggered expansion of *Pea3* expression domain in the spinal cord can be accomplished through several alternative signalling cascades, differentially sensitive to the *Pea3* dosage. Finally, we show that the regulation of MN survival by HGF/Met can equally be achieved *in vitro* and *in vivo* by alternative signalling cascades involving either PI3K-Akt or Src and Mek pathways.

**Conclusions:**

Our findings distinguish MN survival and fate specification, as RTK-triggered responses allowing substitutions of the downstream signalling routes, from nerve growth patterning, which depends on a selective, non-substitutable pathway.

**Electronic supplementary material:**

The online version of this article (doi:10.1186/s12915-014-0056-6) contains supplementary material, which is available to authorized users.

## Background

The assembly of locomotor circuits relies on the parallel development of spinal motor neurons (MNs) and their target muscles, and subsequently, on their coordinated functional wiring. This involves the stepwise acquisition of cell diversification programs in both cell types, each of which leads to the generation of distinct subtypes, the subsequent matching of each motor pool with its cognate target muscle through topographical arrangement of their axonal projections, and finally a process that allows numerical matching of MN pool size to the size of the target muscle [1,2]. During these consecutive phases, a number of aspects of MN biology, including specification, axon growth, guidance and survival, are controlled by multiple target-derived growth factors and their receptors [1]. Most of these growth factors are capable of eliciting multiple responses and the specialisation appears to involve the subtype-specific distribution of their cognate receptors, respectively established through the combinatorial distribution of fate-specifying transcription factors [3]. The resulting combinations of growth factor receptors define a unique set of growth factor responses for each MN subtype, matching either specific muscles or a functional subtype [4,5].

An additional level of complexity appears when considering signalling modalities. The actions of each growth factor receptor upon ligand binding involve the activation of cytoplasmic signalling pathways [6]. The discovery by Tony Pawson of conserved domains in signalling molecules conferring binding ability to activated receptor tyrosine kinases (RTKs) has revolutionised how we see that RTK signalling regulates cellular behaviours. In particular, a profusion of cytoplasmic signalling effectors, binding phosphorylated tyrosines via Src homology domain type 2 (SH2) and type 3 (SH3) and phosphotyrosine binding domain (PTB), can be recruited upon receptor activation by their ligands, to mediate multiple, qualitatively different, biological responses to a given factor. One signalling adaptor can be used as an effector of different biological responses to distinct growth factors. Conversely, a given growth factor receptor can employ distinct signalling effector pathways to mediate distinct cellular responses. Introducing point mutations in the genomic loci of RTKs, such as TrkC, TrkB, Met, Pdgfr or Ret, by selectively modifying the binding sites through which they recruit cytoplasmic effectors, has been instrumental in distinguishing the respective contribution of signalling adaptors, such as PLCγ, Shc, Grb2, PI3K and Src, in mediating qualitatively distinct cellular responses *in vivo* [7–12]. The studies cited above compared cellular behaviours (such as survival, migration, proliferation and target innervation) adopted by cell types with very different biological histories (i.e. neurons versus muscles or vascular smooth muscle cells) but did not distinguish distinct subtypes (molecular or anatomical) within a generic cell type. However, RTK signalling requirements for a given biological response might not always be identical in different cell types or subtypes. Signalling requirements may be determined by the intrinsic competence of each cell type to provide a robust signal transduction platform capable of executing the function, such competence being directly linked to the intrinsic molecular identity of the cell. Thus, to define the robustness of a specific biological outcome requires understanding for a cell type the degree to which several alternative signalling pathways can substitute for each other to execute qualitatively distinct RTK-driven responses, and distinguishing permissive from non-permissive pathways. We chose to address this issue by modulating the signalling competences of a growth factor/RTK system in charge of multiple biological functions in one unique cell type, focusing on MNs, and by exploring specificity versus plasticity of the pathways that intercalate to execute each of these biological responses.

Among the plethora of factors known to influence MN biology, we chose to focus on hepatocyte growth factor (HGF), a multifunctional growth factor whose functions are mediated by the Met RTK. HGF influences MN biology by acting both on MNs and on muscles. Produced by the limb bud mesenchyme, HGF first acts on muscle migration by triggering the delamination of myoblast precursors from limb level somites [13]. This event is necessary for myoblasts to undergo their long-range migration towards the limbs [13–15]. In embryos lacking Met signalling, the absence of limb muscles causes the death of all limb MNs [4,16], most likely by depleting all muscle-derived neurotrophic factors required to support MN survival. Besides this indirect influence, HGF/Met signalling also plays a number of direct functions in MNs, including regulating motor axon growth [4,9,17,18], MN subtype specification [4,16] and MN survival [4,18,19].

Using tissue-specific ablation of the Met receptor, we have recently shown that as development proceeds, HGF acts on distinct MN pools, in which it successively controls several specific biological pathways [4]. On a subset of Met-expressing brachial MNs, HGF acts at early stages (during a muscle-independent period), by influencing cell fate specification (through cell autonomous induction of expression of the transcription factor Runx1 and non-cell autonomous induction of the transcription factor Pea3 in neighbouring neurons) and consequently axon growth toward and within their target muscle (*cutaneous maximus (CM)*) [4,9,16]. After the onset of muscle-dependency for MN survival, highlighted by a peak of naturally occurring cell death (NOCD), which spreads among motor columns to eliminate supernumerary MNs [20], we recently discovered a switch in both the biological response and in the MN pools supported by HGF/Met. As shown with neural-specific ablation of Met after NOCD, HGF/Met signalling is required for the survival of a distinct pool of Met-expressing MNs that innervate the *pectoralis minor* muscle, whereas it is no longer required to support *CM* MNs [4]. Thus, the biological response to a given target-derived growth factor depends both on the MN subtype and on timing, offering an interesting context in which to address robustness versus specificity of signalling requirements.

In this study, we took advantage of an allelic series of *met*, including: a) the *met*^*d*^ signalling dead allele [15]; b) the hypomorphic *met*^*2P*^ and *met*^*2S*^ signalling mutant alleles, in which the multifunctional docking site is converted into optimal binding sites for phosphatidylinositol 3-kinase (PI3K) or Src, respectively [9]; c) an alternative knockout/knock-in *met*^*LacZ*^ allele [4] and d) a conditional *met*^*Flox*^ allele [4,21]. We asked whether execution of three different biological responses (axonal patterning, specification and survival) elicited by the pleiotropic RTK Met in distinct MN subtypes, is compatible with substitutions of the signalling pathways, or requires selective non-substitutable signalling routes. By uncoupling Met functions in MNs from its function in muscle migration, and by selectively allowing Met to signal through either Src or PI3K optimal binding sites, we found that: 1) while HGF/Met signalling indirectly impacts on nerve growth through the regulation of muscle size in the limbs, a direct regulation of motor growth is achieved in the presence of limiting amounts of muscles by activating PI3K, but not Src, pathway; 2) the non-cell autonomous function of HGF/Met that allows expansion of the *Pea3* expression domain in the spinal cord [16], can be accomplished through several alternative signalling cascades, although their competence is differentially sensitive to *Pea3* dosage; 3) regulation of MN survival by HGF/Met can be equally achieved *in vitro* and *in vivo* by alternative signalling cascades involving either PI3K-Akt or Src and Mek pathways.

## Results

**Figure 1:**
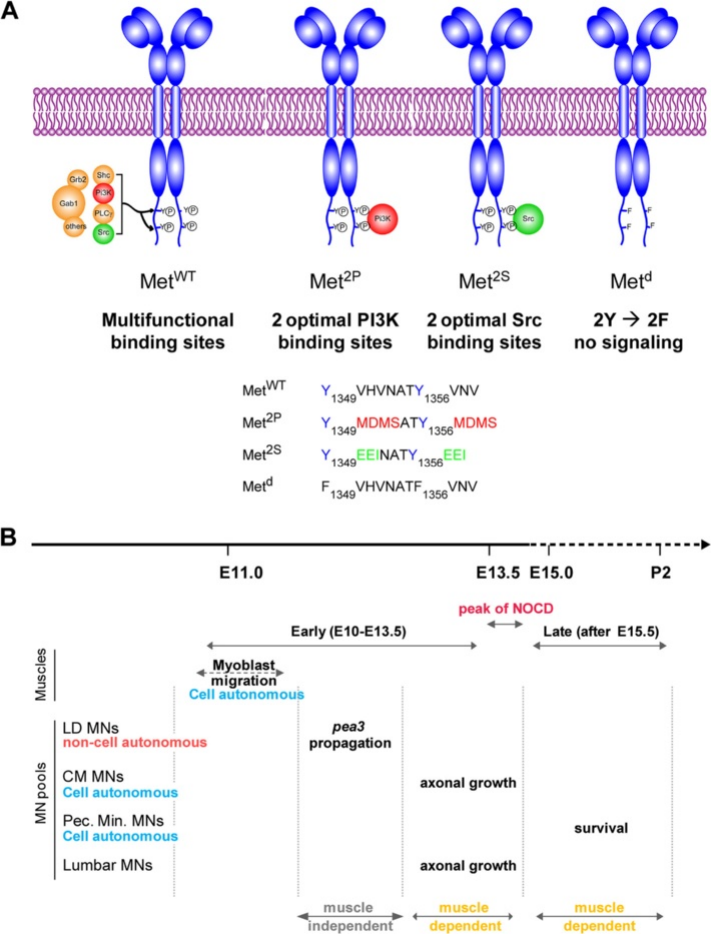
**Using Met specificity-switch mutants to study plasticity of HGF/Met functions in motor neurons. (A)** The wild type (WT) and three signalling mutant versions of the Met receptors used in this study. The WT receptor (Met^WT^) conveys intracellular signalling mainly through two tyrosine residues located in the C-terminal tail. Upon HGF binding and phosphorylation by the Met kinase domain, these tyrosines recruit several cytoplasmic signalling effectors, including Gab1, PI3K, Src, Grb2 and a number of others. The specificity-switch mutants Met^2P^ and Met^2S^ have been designed to carry optimal binding sites for PI3K and Src, so as to favour their respective recruitment/activation [9]. The Met^d^ receptor has its two tyrosines replaced by phenylalanines and is thus signalling incompetent [9,15]. As a consequence, *met* ^*d/d*^ embryos have phenotypes indistinguishable from null mutants. **(B)** Time course of developmental events regulated by HGF/Met signalling that influence MN biology, summarising our earlier findings [4,16]. The period shown is divided into an early period, from E10.5 to E13.5, before synaptic contact between MNs and muscles, and a late period, during which MNs are dependent on muscle-derived trophic factors for their survival. The transition between the two periods is marked by a pronounced peak of MN death (NOCD), indicating numerical adjustment of MN numbers to target muscle size. During the muscle-independent period, before NOCD, HGF/Met signalling controls migration of limb myoblasts, axon growth of a subset of Met-expressing MNs, cell autonomous induction of *runx1* [4] and non-cell autonomous propagation of *Pea3*-expression [16]. After NOCD, HGF/Met controls the survival of a distinct pool of MNs, innervating the *pectoralis minor* muscle, while it is no longer required for trophic support of CM or *latissimus dorsi* (LD) MN pools.

**Figure 2:**
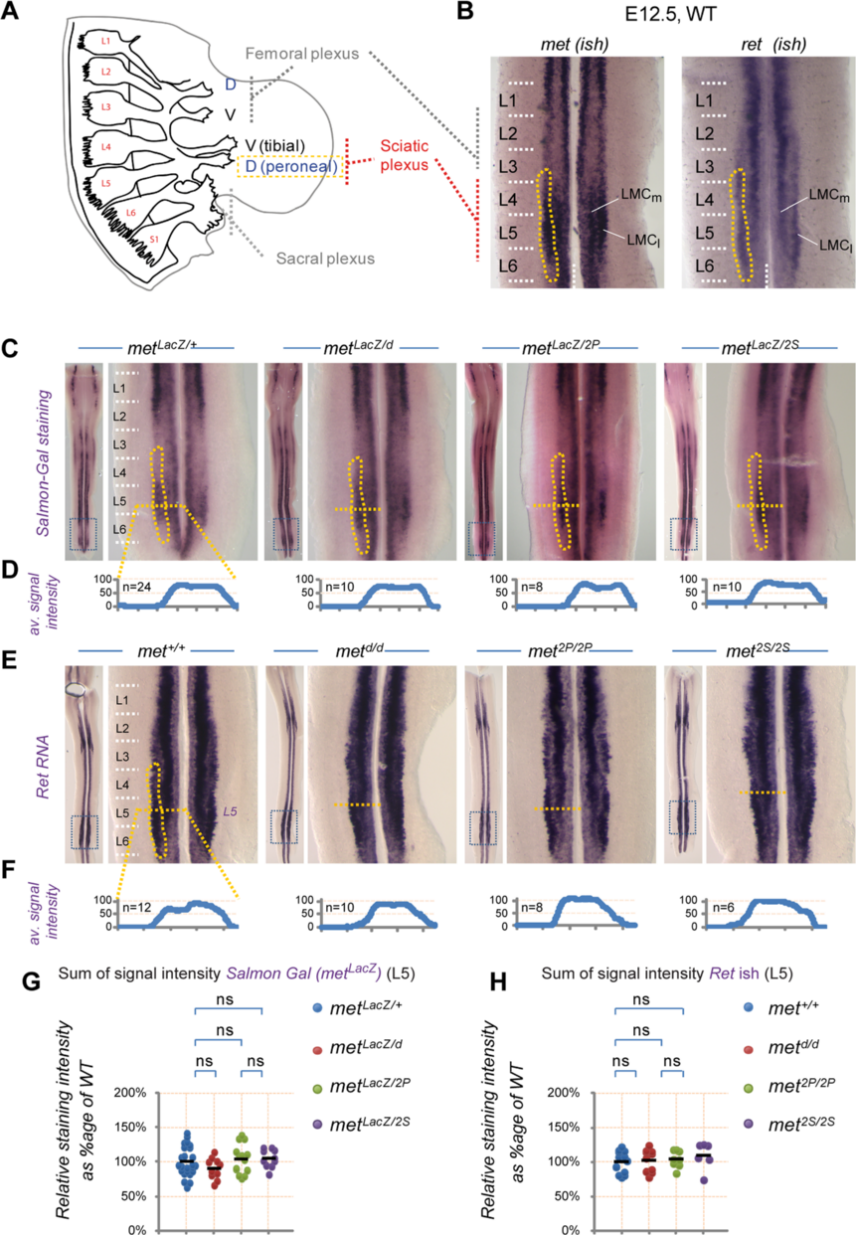
**Met signalling is required for hindlimb nerve patterning prior to the onset of muscle-dependency for MN survival. (A)** Lumbar spinal nerves and plexuses at hindlimb levels based on a lateral view of an E11.5 WT embryo. The sciatic plexus (from which the tibial (ventral) and peroneal (dorsal) nerves emerge) receives axons mostly from L4 and L5 spinal segments, while the femoral plexus (anterior) collects axons mostly from L2 and L3 spinal segments. **(B)** ISH with *Met* and *Ret* probes on E12.5 WT spinal cords. The LMC pool corresponding to peroneal MNs is indicated in yellow, spanning from mid L3 to L6. **(C)** *Met* expression was followed by Salmon-Gal staining in spinal cords from E12.5 *met* ^*LacZ/+*^, *met* ^*LacZ/d*^, *met* ^*LacZ/2P*^, and *met* ^*LacZ/2P*^ embryos. The position of peroneal MNs is indicated in yellow. **(D)** Quantification of the size of Met-expressing MN pools along the orange line at L5 level. Each plot represents the average signal distribution of the indicated number of spinal cord sides (*met* ^*LacZ/+*^: n=24; *met* ^*LacZ/d*^: n=12; *met* ^*LacZ/2P*^: n=8; *met* ^*LacZ/2P*^: n=10. **(E)** Ret expression was followed by ISH in lumbar spinal cords from E12.5 WT, *met* ^*d/d*^, *met* ^*2P/2P*^, *met* ^*2S/2S*^ embryos. **(F)** The size of the Ret-expressing MN pools was quantified along the orange line at L5 level. Each plot represents the average signal distribution of the indicated number of spinal cord sides (*met* ^*+/+*^: n=12; *met* ^*d/d*^: n=10; *met* ^*2P/2P*^: n=8; *met* ^*2S/2P*^: n=6). **(G,** **H)** Quantifications and statistical analyses of the sum of signal intensity corresponding to measurements of *met* ^*LacZ*^ expression **(G)** or Ret ISH staining **(H)** based on intensity plots in **(D)** and **(F)**, respectively. The numbers of samples are as indicated in **(D)** and **(F)**. At E12.5 the size of the MN population is not significantly altered by the reduced muscle mass in *met* ^*d/d*^, *met* ^*2P/2P*^, and *met* ^*2S/2P*^ embryos, indicating that this stage precedes the phase of MN death.

Most signalling by the Met receptor is mediated by two C-terminal tyrosines, referred to as multifunctional docking sites. The surrounding amino-acid environment (Y_1349_VHVNATY_1356_VNV) is permissive to the binding of multiple signalling adaptors (Figure 1A) [22]. Whereas converting both tyrosines to phenylalanine completely abrogates signal transduction (Met^d^ signalling dead receptor [15]), modifying the amino-acids surrounding these tyrosines can alter the selective affinity for some cytoplasmic effectors (Figure 1A) [9]. By converting the two docking sites into optimal binding sites for PI3K (Y_1349/1356_MDMS) or for Src (Y_1349/1356_EEI), the Met^2P^ and Met^2S^ receptors, respectively, were designed to determine the impact of imposing the cytoplasmic effector through which Met can signal, thus raising its contribution above generic signalling levels (Figure 1A). These specificity-switch mutants have been instrumental in addressing the relevance of selective pathways in several biological contexts [9,23–26]. Regarding the function of HGF/Met that triggers myoblast delamination and migration towards limb buds [13–15], the Met^2P^ and Met^2S^ specificity-switch mutants have limited ability to elicit long-range myoblast migration (Additional file 1: Figure S1A) [9]. In spite of this, the small number of migrating myoblasts is nevertheless sufficient to account for the formation of a substantial amount of muscle in both forelimbs and hindlimbs (Additional file 1: Figure S1A and Figure 2B), and does not affect MN numbers prior to the onset of muscle dependency for survival (Figure 2: lumbar MNs; Additional file 1: Figure S1B: brachial MNs) [16]. This allelic series of *met* mutants thus represents an excellent toolbox to uncouple the effects of loss of muscle from direct functions of Met in MNs. Here, we took advantage of the differences in signalling competences of these two versions of Met to determine the degree of flexibility versus specificity for a given biological outcome, such as those elicited by HGF/Met in selective subsets of Met-dependent MN pools (Figure 1B).

### Distinct signalling requirement for nerve patterning

HGF, initially provided by the limb mesenchyme and later by the muscles themselves [19,27,28], is known to promote growth, to exert chemoattractant activity [17,18] and to pattern nerve branching of motor axons [12,17,18]. However, a number of abnormalities in peripheral nerve patterns observed in *met* or *hgf* loss-of-function mutants [12,17,18], result from secondary effects of the absence of muscles, from the subsequent depletion of muscle-derived guidance signals and from early changes in MN specification [16]. Thus, in spite of extensive documentation of constitutive knockout phenotypes [9,17,18] and our own conditional studies bypassing requirements prior to NOCD [4], formal *in vivo* demonstration of a direct requirement for Met in axon growth and path-finding is still currently lacking. We have previously shown that neural-specific Met excision with a Nestin-cre driver was only complete by the end of the NOCD period [4], preventing use from addressing the possibility of a direct requirement for axon growth and guidance prior to NOCD using a conditional approach. Thus, our paradigm using the specificity-switch mutants is suitable for addressing this question.

We previously reported that HGF/Met signalling influenced axon growth in the *CM* muscle [9]. However, guidance of *CM* MN axons to their target does not reflect a direct function of HGF/Met in axon growth, as it is conditioned by an early specification program establishing *Pea3* expression domain in a MN pool, which is under the control of Met as well (see later). To circumvent these caveats and examine signalling modalities of HGF/Met in a system where they exert direct control of axonal growth and guidance, we chose to investigate the motor nerve pattern in the dorsal hindlimb. While *Met* expression in brachial spinal cord segments is restricted to the *Pea3*-expressing MN pool, in which HGF/Met controls cell fate specification, *Met* is also expressed in pools of lumbar MNs, including medial and lateral LMC MN columns at sciatic levels L4 to L6, in which *Pea3* is not co-expressed (Figure 2A,B) [19]. We first established that at E12.5, prior to the onset of cell death caused by trophic deprivation, no difference in the amount of *Ret*-expressing MNs in the lumbar segments could be detected in any of the *met* mutant genotypes (*met*^*d/d*^, *met*^*2P/2P*^ and *met*^*2S/2S*^) compared to controls (Figure 2E,F,H). Further confirmation came from analysis of *met*-expressing neurons directly, by following one copy of the *met*^*LacZ*^ reporter/knockout allele, in *met*^*LacZ/+*^, *met*^*LacZ/d*^, *met*^*LacZ/2P*^ and *met*^*LacZ/2S*^ embryos (Figure 2C,D,G).

**Figure 3:**
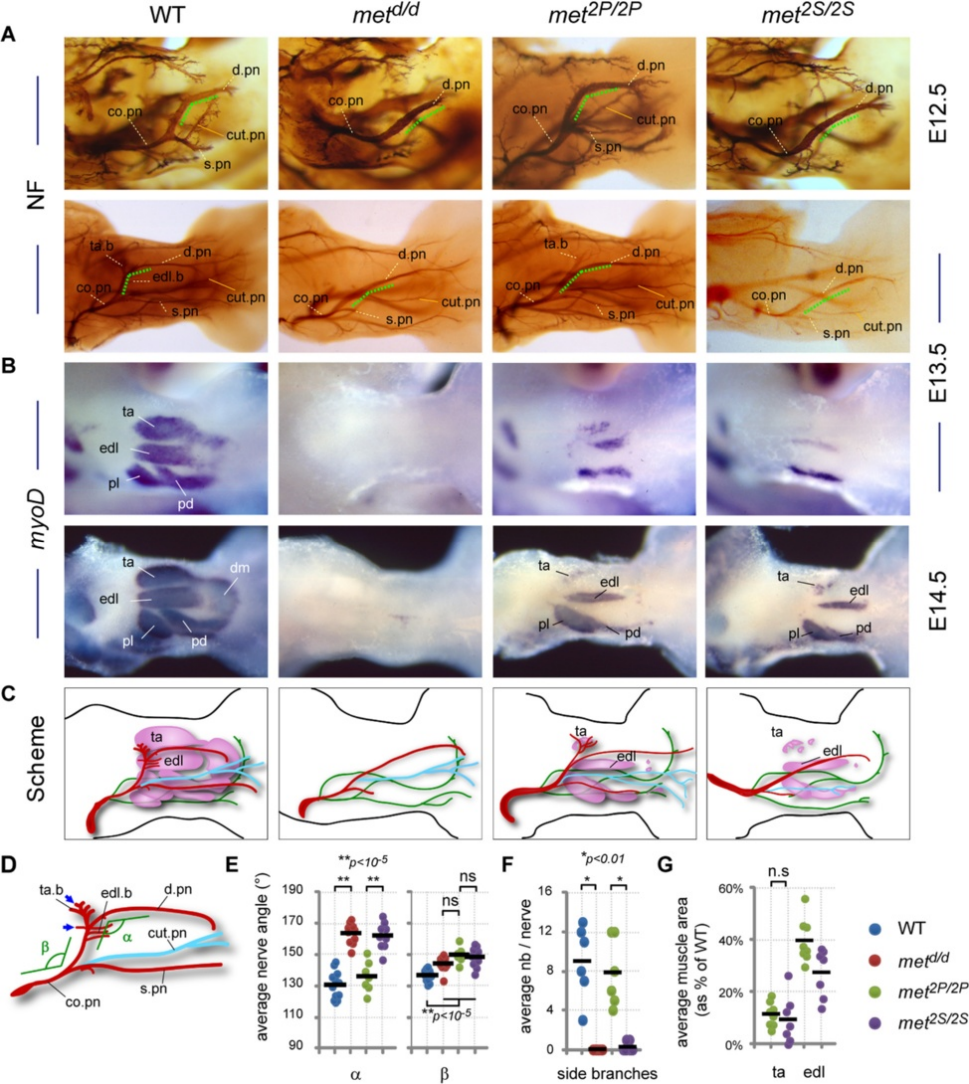
**Uncoupling muscle-dependent from Met-dependent axonal branching. (A)** Anti-neurofilament (NF) immunohistochemistry on E12.5 and E13.5 hindlimbs of WT and *met* mutants. **(B)** *MyoD* ISH showing differentiating muscles in E13.5 and E14.5 hindlimbs. **(C)** Recapitulative schemes positioning hindlimb muscles and nerves with respect to each other. Colour code for nerves; green: ventral nerves not in the focal plane and unaffected by lack of Met signalling; red: peroneal nerve motor branches, innervating the dorsal limb compartment, corresponding to the common peroneal nerve as it emerges from the sciatic plexus. The cutaneous sensory peroneal nerve branch is shown in blue. **(D)** Quantification parameters. α: angle between the transverse and distal portion of the deep peroneal nerve. β: angle between common and deep peroneal nerve portions, used as internal morphological landmark. Blue arrows: contact sites with ta and edl muscles where the number of peroneal nerve side branches were quantified. **(E)** The nerve phenotype severity was assessment by measuring the α and β angles, in WT (n=12), *met* ^*d/d*^ (n=10), *met* ^*2P/2P*^ (n=7), and *met* ^*2S/2S*^ (n=9) E12.5 and E13.5 hindlimbs. Each dot represents one embryo side. The same colours representing the genotypes were used in all graphs. **(F)** Plot showing the respective numbers of side-branches per peroneal nerve (pooling ta and edl branches), in E13.5 WT (n=6), *met* ^*d/d*^ (n=7), *met* ^*2P/2P*^ (n=6), and *met* ^*2S/2S*^ (n=8) hindlimbs. Differences between WT and *met* ^*d/d*^ embryos, and between *met* ^*2P/2P*^ and *met* ^*2S/2S*^ embryos, respectively, are significant (Mann-Whitney test, p<0.01).) **(G)** Measurements of ta and edl muscle surface were performed on WT (n=6), *met* ^*d/d*^ (n=6), *met* ^*2P/2P*^ (n=8), and *met* ^*2S/2S*^ (n=7) E13.5 and E14.5 hindlimbs stained with *MyoD* ISH. Areas were expressed as percentage of the mean area in WT embyos (represented as 100%). The plot therefore shows only *met* ^*2P/2P*^ and *met* ^*2S/2S*^ values, each dot representing one embryo side.

We next performed careful correlative analysis of nerve and muscle patterns in the dorsal hindlimb, using whole-mount anti-neurofilament staining and *MyoD in situ* hybridisation (ISH). In WT embryos, the dorsal shank is mainly innervated by the peroneal nerve branch emerging from the sciatic plexus (Figure 2A) [29]. This nerve follows a stereotyped trajectory, first crossing the *extensor digitorum longus* (EDL) muscle on its proximal extremity, and continuing its distal progression towards the foot in the space between the *EDL* muscle and the *tibialis anterior* (TA) muscle (see scheme in Figure 3D). In wild-type (WT) embryos, the sharp turn made by the peroneal nerve between the portion crossing the *EDL* muscle and the longitudinal portion running between *EDL* and *TA* muscles consistently defines an angle (α) of approximately 120° on average. Furthermore, at the contact site between the nerve and each muscle, a few axonal side branches leave the nerve to enter the muscle where they will later establish synapses with muscle fibres (Figure 3D, blue arrows, see also Additional file 2: Figure S2E). In *met*^*d/d*^ embryos, in the absence of limb muscles, the peroneal nerve displayed no detectable side branches and a significantly altered shape (Figure 3A,C), running straight through the limb with α increased to approximately 160° on average (Figure 3E, **P < 0.001). Although we found reduced amounts of *Pea3*-expressing MNs in the lumbar region (Additional file 2: Figure S2C), this peroneal nerve phenotype is not a consequence of altered *Pea3* expression, as: (a) *Pea3* is not co-expressed with *met* in peroneal MN pools at L4–L5 levels (yellow dotted pools, Additional file 2: Figure S2B), and (b) *Pea3*-null mutants exhibit an intact peroneal nerve shape (Additional file 2: Figure S2D). Thus, the peroneal nerve shape is an appropriate model for unambiguously establishing Met-signalling requirements for axonal growth, without possible consequences related to defects in muscle formation and/or MN specification.

We first established a cartography of developing muscles in *met*^*2P/2P*^ and *met*^*2S/2S*^ dorsal hindlimbs and found an asymmetric pattern, with the posterior muscles (including *peroneus longus* and *peroneus digiti*) showing a reduced mass but almost normal shape, while muscles of the anterior group (including *EDL* and *TA* muscles) were severely reduced in size, and the distal muscle mass was completely absent (Figure 3B,C,G). The anterior-most muscle, *TA,* was the most affected, and its range of phenotypical variations included a complete absence in a small subset of embryos. The overall quantification of its size revealed no significant difference between *met*^*2P/2P*^ and *met*^*2S/2S*^ embryos. There was also no significant difference in the size of the *EDL* until E14.5, when secondary myogenesis starts [30]. While examining the nerve pattern, we found that the peroneal nerve of *met*^*2P/2P*^ embryos had a shape similar to that of control embryos, with an average α of approximately 135°, and its side branches in contact with *EDL* and *TA* were present in most cases (with an average number of eight side branches per nerve compared to nine per nerve in controls; Figure 3F). In contrast, the peroneal nerve shape of *met*^*2S/2S*^ embryos was identical to that seen in *met*^*d/d*^ mutants, with an average α of approximately 160° and side branches towards *EDL* and *TA* never detected. Of notice, another internal morphological landmark, β, reflecting the turn made by the peroneal nerve emerging from the sciatic nerve, did not exhibit significant differences between *met*^*d/d*^, *met*^*2S/2S*^ and *met*^*2P/2P*^ embryos, although significantly affected by the morphological changes in muscle shape compared to controls. Altogether, these data demonstrate that signalling by Met^2P^, but not by Met^2S^, can efficiently promote side-branch formation, and shape the peroneal nerve, provided that the corresponding muscle is present in the limb (likely to supply either HGF itself or a cofactor necessary for the growth response). Furthermore, they indicate that distinct signalling cascades, such as those triggered by these two receptor variants, are not equivalent in their capacity to execute nerve patterning by Met.

### Signalling cascades effective at mediating Met-triggered *Pea3*-domain expansion are differentially sensitive to *Pea3* gene dosage

**Figure 4:**
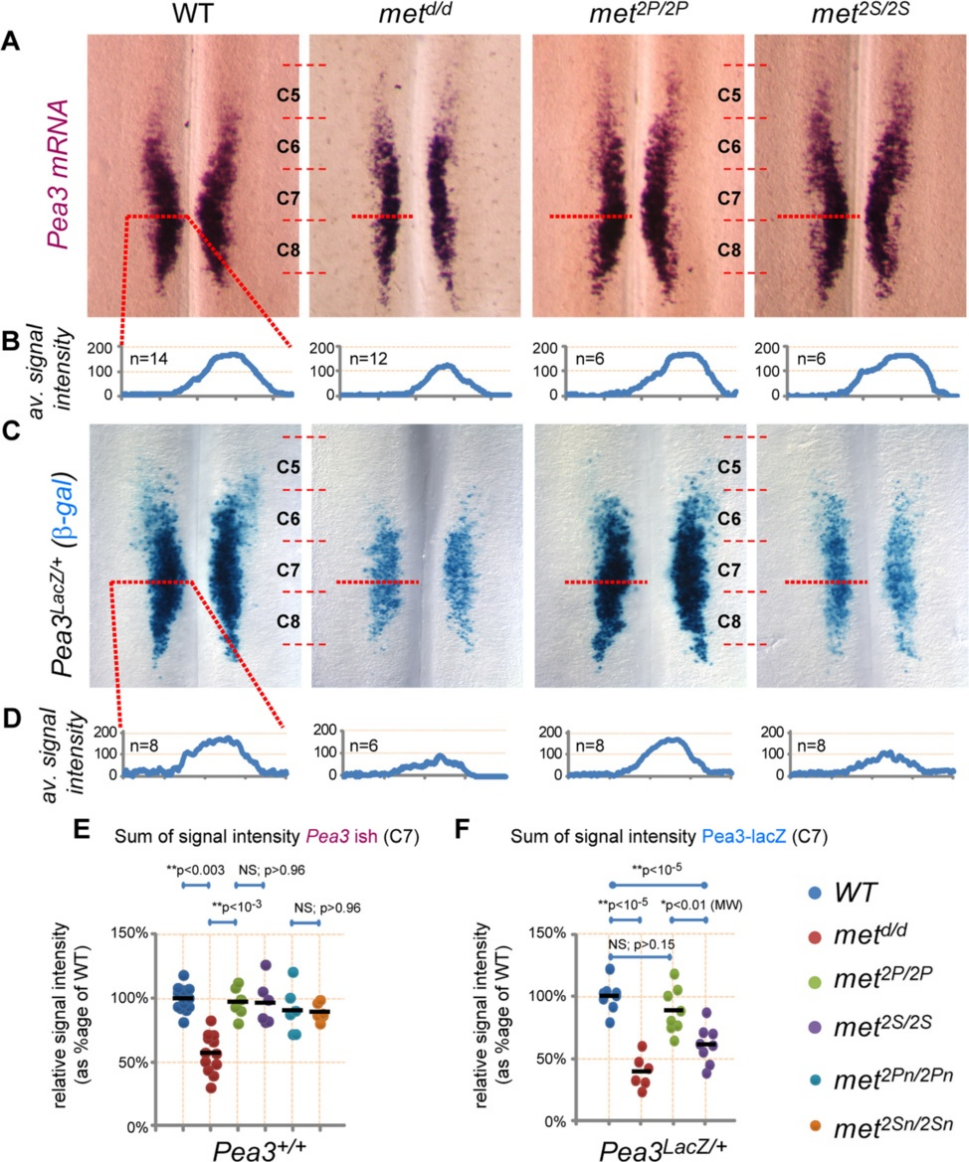
**Met-induced** ***Pea3*** **propagation can be executed through PI3K and Src pathways. (A)** ISH analysis of the establishment of *Pea3* expression domain in spinal MNs . Images show brachial region of flat-mounted E12.5 spinal cords hybridised with *Pea3* probes in WT, *met* ^*d/d*^, *met* ^*2P/2P*^, and *met* ^*2S/2S*^ embryos. Whereas *Pea3* MN pool is reduced in *met* ^*d/d*^, it appears normal in *met* ^*2P/2P*^ and *met* ^*2S/2S*^ embryos. **(B)** Quantifications of the lateral expansion of the *Pea3* domain by signal intensity analysis: each plot represents the average signal distribution measured on the indicated number of spinal cord sides along the red dotted line as positioned in each image in **(A)** (WT: n=14; *met* ^*d/d*^: n=12; *met* ^*2P/2P*^: n=6; *met* ^*2S/2S*^: n=6). **(C)**: Brachial regions of flat mounted spinal cords stained with X-Gal to reveal β-galactosidase activity of the *Pea3* ^*LacZ*^ knock-in. This context with only one functional copy of *Pea3* is sufficient for complete *Pea3* propagation by Met^2P^, but is not permissive for Met^2S^ receptor. There was a lack of propagation in *met* ^*d/d*^: *Pea3* ^*LacZ/+*^ as compared to *met* ^*+/+*^:*Pea3* ^*LacZ/+*^ spinal cords. **(D)** Quantifications of the lateral expansion of the *Pea3*/β-galactosidase-positive domain by signal intensity analysis: each plot represents the average signal distribution as measured on the indicated number of spinal cord sides (WT: n=8; *met* ^*d/d*^: n=6; *met* ^*2P/2P*^: n=8; *met* ^*2S/2S*^: n=8) along the red dotted line as positioned in each image in **(C)**. **(E,F)** Quantifications and statistical analyses of the sum of the signal intensities corresponding to measurements of *Pea3* ISH in a *Pea3* ^*+/+*^ context **(B)**, or for X-gal staining in a *Pea3* ^*LacZ*^ context **(D)**. Each dot represents the sum of intensity for one sample, the number of samples being as indicated in **(B)** and **(D)**. The absence of one *Pea3* functional copy in a *Pea3* ^*LacZ*^ context reveals a significant difference in *Pea3* dosage requirement between *met* ^*2P/2P*^ and *met* ^*2S/2S*^ embryos.

We next investigated the signalling requirement for cell fate specification by focusing on the program that defines which MN pools express the transcription factor Pea3, in which HGF/Met signalling is required [16]. We have previously shown that the *Pea3*-expressing MN population at brachial levels is constituted of two main subgroups of MNs [16]. In the first group of pioneer MNs, *Pea3* expression is initially induced by glial-derived neurotrophic factor (GDNF), produced by the target muscle [31]. HGF then acts on the same pioneer MNs, via Met, instructing them to induce *Pea3* expression in additional neurons, a feed-forward induction process that leads to lateral and anterior expansion of the *Pea3* expression domain (summarised in Additional file 3: Figure S3, see also [16,31]). Such biphasic establishment of the *Pea3* expression domain is illustrated by the phenotype of *met*^*d/d*^ mutants, in which this second process is abolished, leaving only the GDNF-dependent population [16]. Importantly, the size reduction of *Pea3*-expressing motor pools observed in *met*^*d/d*^ mutants occurred before the onset of muscle dependency and was not caused by muscle depletion, since such a defect was not observed in Pax3-deficient (*Pax3*^*Sp/Sp*^) embryos, which also lack limb muscles [16]. Whole-mount ISH with *Pea3* on E12.5 spinal cords revealed the *Pea3* expression domain in *met*^*2P/2P*^ and *met*^*2S/2S*^ spinal cords had a normal shape, contrasting with the reduced shape displayed in *met*^*d/d*^ mutants (Figure 4A,B,E). We ruled out that the competence of Met^2P^ and Met^2S^ signalling mutants to mediate expansion of *Pea3* expression domain could be indirectly influenced by the reduced muscle content, by showing that: (a) no detectable changes in the amount of *Ret*-expressing MNs were found in E12.5 *met*^*d/d*^, *met*^*2P/2P*^ and *met*^*2S/2S*^ brachial spinal cords (Additional file 1: Figure S1B); (b) the *Pea3* domain expansion is independent of muscle-derived signals, and occurs at a stage when the muscle phenotype of *met*^*d/d*^, *met*^*2P/2P*^ and *met*^*2S/2S*^ embryos is almost equivalent (E11.5) (Additional file 1: Figure S1A; see also [16]); (c) comparably sized *Pea3* and *Ret* expression domains (Additional file 4: Figure S4B,C and Figure 4B) were also observed in the brachial spinal cord of *met*^*2Pneo/2Pneo*^ and *met*^*2Sneo/2Sneo*^ signalling-switch mutant embryos still carrying the *neo* cassette (which had served to engineer the modified alleles, the latter causing attenuated expression levels of the mutant receptors [9] and resulting in further reduced muscle masses (Additional file 4: Figure S4A)). Thus, the signalling routes used by the Met^2P^ and Met^2S^ specificity-switch mutants are equally sufficient, even when sensitised by lowered receptor expression levels, to execute the mechanisms leading to *Pea3* propagation.

The signalling cascades triggered by the Met^2P^ and Met^2S^ specificity-switch mutants involve distinct, non-equivalent mechanisms [9]. However, besides the selective activation of PI3K and Src pathways, the two receptors also share a common set of signalling effectors, such as the adaptor molecule Gab1 and its downstream signalling components [9], which could account for their equivalent capacity to induce *Pea3* propagation. To discriminate between activation of selective pathways versus background levels of shared downstream effectors, we asked whether the signalling mechanisms in *met*^*2P/2P*^ and *met*^*2S/2S*^ embryos could be genetically distinguished. We reasoned that a way to address this issue was provided by the fact that the process of expansion of *Pea3* expression involves a positive reinforcement loop during which *met* and *Pea3* are necessary for each other’s expression (see Additional file 3: Figure S3) [16]. Indeed, we have shown that *Pea3* itself contributes to its own propagation, since it is necessary to induce *Met* expression within the pioneer domain, as shown in *Pea3*^*-/-*^ mutants [16]. Consistently in *met* loss-of-function mutants, the reduced expansion of the *Pea3* domain also results in reduced *met* expression, as assessed by comparing β-galactosidase activity in *met*^*LacZ/+*^ and *met*^*LacZ/d*^ embryos (Additional file 1: Figure S1D,G), whereas expansion of *met*^*LacZ*^ expression was fully executed in *met*^*LacZ/2P*^ and *met*^*LacZ/2S*^ embryos, paralleling the intact expansion of *Pea3* expression domain (Additional file 1: Figure S1D,G). We therefore challenged the robustness of the MN pool specification function mediated by the *Met* signalling-switch mutants, by genetically lowering *Pea3* expression levels. We took advantage of the *Pea3*^*LacZ*^ knockout allele, in which the insertion of the *LacZ* gene not only results in loss of *Pea3* functions, but also recapitulates the *Pea3* expression domain [32]. Intriguingly, we found that whereas *met*^*2P/2P*^*; Pea3*^*LacZ/+*^ had a pattern of β-galactosidase activity similar to *Pea3*^*LacZ/+*^ control E12.5 brachial spinal cords, *met*^*2S/2S*^*; Pea3*^*LacZ/+*^ displayed a significantly reduced number of blue MNs, resulting in a phenotype similar to that of *met*^*d/d*^*; Pea3*^*LacZ/+*^ mutants (Figure 4C,D,F). Thus although switching signalling cascades activated by Met mutants leads to comparable efficacy to expand the *Pea3* expression domain in the context of two functional copies of *Pea3*, the signalling routes employed by the Met signalling variants are differentially sensitive to *Pea3* gene dosage reduction: the Met^2S^ route is sensitive, whereas the Met^2P^ is not. This indicates that higher *Pea3* levels are required for its own propagation of expression when mediated through a Met^2S^/Src-dependent mechanism, whereas lower *Pea3* doses are sufficient when propagation occurs via a Met^2P^/PI3K-dependent cascade. Thus, the program for acquisition of Pea3 identity triggered by HGF/Met allows a certain degree of signalling flexibility, and the competence of alternative pathways to execute this function is differentially dependent on the genetic dosage of *Pea3*.

### Plasticity of Met signalling requirement for a cell autonomous survival response in primary motor neuron cultures

We next explored the plasticity of the MN survival supporting function of HGF/Met when challenged with signalling switches. HGF is known to promote the survival of subsets of limb-level MNs in culture [4,18,19]. *In vivo*, the HGF/Met requirement for MN survival is limited to lumbar MNs in chicks [33] and to a selective pool of brachial MNs innervating the *pectoralis minor* muscle in mice, as shown with neural-specific ablation of Met [4], prompting us to focus our studies on this MN pool. Studying the dependency on HGF/Met for MN survival requires distinguishing its direct trophic function from the indirect consequences of the trophic factors for depletion resulting from myoblast migration defects. Bypassing the consequences of decreased muscle mass in the limbs of *met*-signalling mutant embryos can be achieved *in vitro* using primary MN cultures and *in vivo* through neuronal lineage-restricted signalling modulation of Met properties.

**Figure 5:**
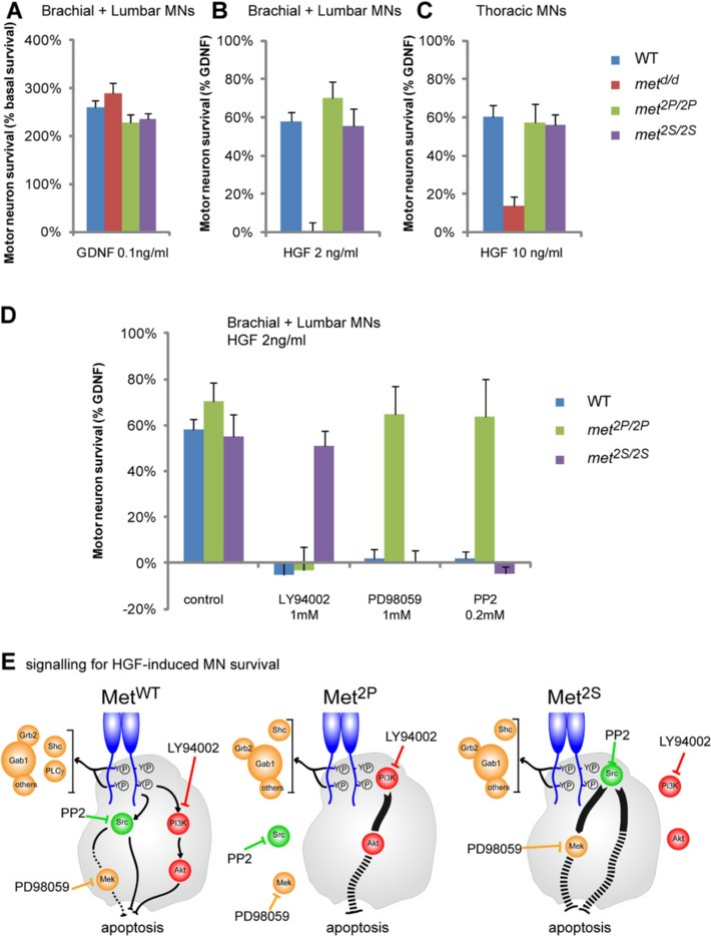
**Cell autonomous regulation of MN survival by Met can be achieved through distinct signalling pathways. (A)** *In vitro* assessment of survival of MNs derived from WT, *met* ^*2P/2P*^, *met* ^*2S/2S*^, and *met* ^*d/d*^ E12.5 embryos in the presence of GDNF (100 pg/ml). Survival values are expressed as percentage of the survival in basal medium (defined as 100%). The ability of MNs to respond to GDNF is not altered in *met* ^*d/d*^, *met* ^*2P/2P*^, and *met* ^*2S/2S*^ mutants. Therefore, in subsequent panels, values are normalised with the survival in basal medium (defined as 0%) and expressed as percentage of their response to GDNF (defined as 100%). **(B,C)** Survival of brachial + lumbar **(B)** or thoracic **(C)** MNs derived from WT, *met* ^*2P/2P*^, *met* ^*2S/2S*^, and *met* ^*d/d*^ E12.5 embryos in the presence of HGF (2 ng/ml). Whereas *met* ^*d/d*^ MNs do not show any survival response to HGF, *met* ^*2P/2P*^ and *met* ^*2S/2S*^ MNs respond as efficiently as WT MNs. **(D)** Signalling requirements downstream of Met^WT^, Met^2P^, and Met^2S^ for MN survival by HGF were explored using inhibitors of PI3K (LY294002: 1 μM), Mek (PD98059: 1 μM), and Src (PP2: 0.2 μM), respectively). Inhibitors were used at concentrations not toxic on the basal survival. All three inhibitors efficiently blocked the survival response of WT MNs to HGF. As expected, the survival response in *met* ^*2P/2P*^ MNs was selectively blocked by the PI3K inhibitor. In contrast, the survival response in *met* ^*2S/2S*^ MNs was abolished by inhibiting Src or Mek. For each genotype, two to four independent experiments were done in triplicate comparing MNs from mutant embryos and from their WT littermates. (WT: n=9; , *met* ^*2P/2P*^: n=3; *met* ^*2S/2S*^: n=4; *met* ^*d/d*^: n=2). Error bars indicate standard error of the mean. **(E)** Schematic representation of the signalling components involved in blocking apoptosis downstream of the wild-type Met^WT^ or the specificity switch mutants Met^2P^ and Met^2S^ receptors. The inhibitors used are indicated.

As a first approach, we assayed survival in primary MN cultures established as described [34] from WT or *met*-signalling mutant embryos at E12.5, a stage when MN numbers were not affected yet by reduced muscle mass (see Figure 3 and Additional file 1: Figure S1B). E12.5 primary MNs were collected and pooled from brachial and lumbar spinal cords, and then cultured in the presence of either GDNF (100 pg/ml) or HGF (0.4 ng/ml or 2 ng/ml) for 3 days. As previously reported for rat MNs, both factors prevented the death of a proportion of WT limb innervating MNs (Figure 5A,B: WT) [4,19,35]. Since MNs derived from *met*^*d/d*^, *met*^*2P/2P*^, and *met*^*2S/2S*^ embryos retained their ability to respond to GDNF (Figure 5A) and to BDNF (data not shown), the response to HGF was expressed as a percentage of GDNF-induced survival, offering the possibility to normalise all experiments performed. While *met*^*d/d*^ MNs no longer responded to HGF, MNs derived from *met*^*2P/2P*^ and *met*^*2S/2S*^ mutants exhibited a survival response to HGF similar to that of WT embryos (Figure 5B). As reported for the rat, WT thoracic and sacral MNs required a higher concentration of HGF (10 ng/ml) to survive [19]. We found that MNs purified from thoracic and sacral regions (non-limb-innervating MNs) from *met*^*2P/2P*^ and *met*^*2S/2S*^ but not from *met*^*d/d*^ embryos survived in response to HGF treatment (data not shown). Since at the stage at which MNs are collected for these primary cultures (E12.5) most Met-expressing MNs are equally present in all genotypes, (Figure 3 and Additional file 1: Figure S1), the absence of a survival response by *met*^*d/d*^ mutant MNs is not a consequence of a complete depletion of *met*-positive neurons, but indicates that the Met^d^ receptor does not transduce survival signals. In contrast, cultures from *met*^*2P/2P*^ and *met*^*2S/2S*^ spinal cords started with an unaltered Met-responsive population compared to WTs (Figure 3, Additional file 1: Figure S1, Additional file 5: Figure S5). As their survival in response to HGF was comparable to survival of WT MNs, this indicates that the Met^2P^ and Met^2S^ receptors mediate the survival induced by HGF as efficiently as the WT receptor. Altogether, these results establish that signalling routes solicited by Met^WT^, Met^2P^ or Met^2S^ are equally capable of triggering *in vitro* MN survival by HGF.

The ability of the Met^2P^ receptor to mediate a survival response to HGF can be understood as the recruitment and activation of the PI3K-Akt pathway, which is well known to be involved in the inhibition of apoptosis [36]. In contrast, the survival function triggered by Met^2S^ suggests a novel link between Src and a survival pathway in MNs. We therefore asked whether activation of Src mediated the survival response either by converging onto the PI3K-Akt pathway (as reported for GDNF-induced neuronal survival [10]), or by acting through an independent mechanism. This question was addressed by pharmacologically inhibiting the PI3K pathway or the Src family kinases with the selective inhibitors LY294002 or PP2, respectively, at concentrations that do not present toxic effects on the basal survival (LY294002: 1 μM; PP2: 0.2 μM; data not shown). We found that the survival responses mediated by Met^2P^ or by Met^2S^ were selectively abolished by LY294002 and by PP2, respectively (Figure 5D), but not by PP3, a non-functional analogue of PP2 (data not shown). These findings argue that the MN survival response triggered by Met^2P^ or Met^2S^ specificity-switch mutants are indeed mediated by PI3K or Src, respectively, as they were designed to do [9]. Using PP2 and LY294002, we further showed that signalling by Met^2P^ does not require Src activity for survival, whereas the survival response elicited by Met^2S^ does not require PI3K activity (Figure 5D). Interestingly, pharmacological inhibition of Mek by PD98059 prevented the survival response elicited by Met^2S^, but not by Met^2P^ (Figure 5D). Together, these experiments indicate that the MN survival promoted by these specificity-switch receptors occurs through activation of distinct sets of signalling pathways. The survival response triggered by Met^2P^ in MNs is mediated through an efficient activation of PI3K, and required neither Src family kinases nor the Mek pathway. In contrast, the mechanism by which Met^2S^ mediates the survival response does not involve the traditional PI3K-Akt survival pathway, but rather activation of both Src family kinases and the Mek pathway. Intriguingly, we found that all three inhibitors abolished the survival response to HGF by WT Met in MNs (Figure 5D). This indicates that unlike the mutant receptors, for which altered docking sites have forced the choice of one respective signalling pathway, in a WT context, Met uses a combination of these three signalling pathways. Altogether, the combination of our genetic and pharmacological approaches indicates that *in vitro* there is a remarkable plasticity in the signalling requirements for MNs survival, which can be achieved equally efficiently by alternative pathways permissive for promoting survival (Figure 5E).

### Plasticity of signalling effectors for Met-dependent motor neuron survival *in vivo*

**Figure 6:**
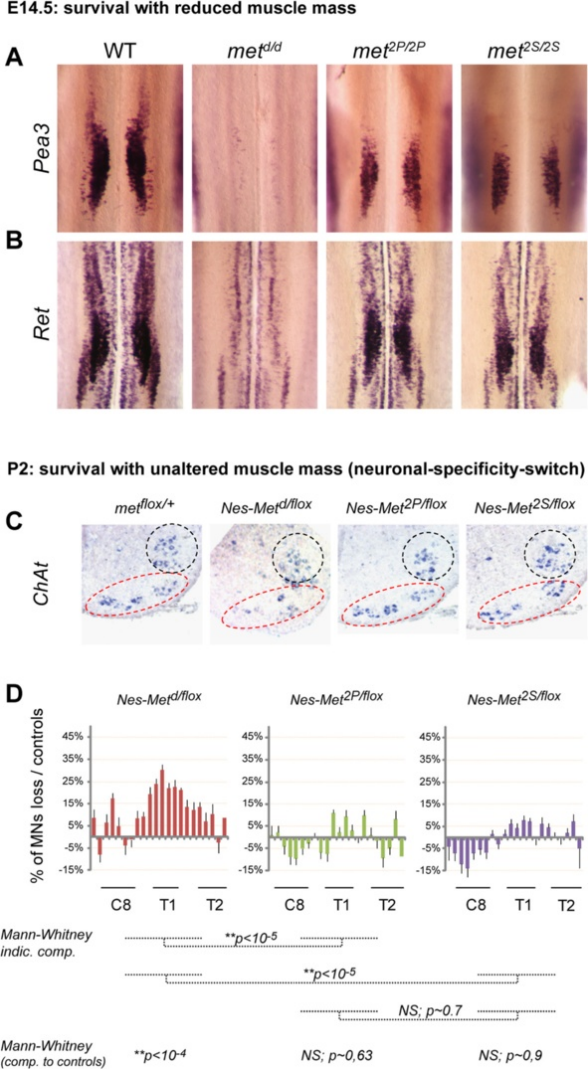
**Conditional neuronal-specificity-switch of Met signalling to PI3K and Src is sufficient for** ***pectoralis minor*** **motor pool survival** ***in vivo*** **. (A,B)** Correlation between MN numbers in spinal cords with the amount of limb muscle in Met signalling mutants (see Figure 2A,B). Analysis of MN pool survival *in vivo* after the onset of muscle dependency by *Pea3* **(A)** or *Ret* **(B)** ISH on spinal cords from E15.5 WT, *met* ^*d/d*^, *met* ^*2P/2P*^, and *met* ^*2S/2S*^ embryos (n = 3 spinal cords for each probe and each genotype). Limb muscle depletion in *met* ^*d/d*^ embryos causes the death of all limb-innervating MNs, hence the complete loss of *Pea3* ^+^ neurons and *Ret*-positive LMC but not axial columns. **(C,** **D)** Analysis of MN survival in *Nestin-Cre* (referred to as *Nes*) mediated neuronal-specificity-switch mutants, which display unaltered amounts of muscle. **(C)** High magnification views of *ChAt* ISH staining of the ventral horn of spinal cord cross-sections in *Nes-Met* ^*flox/+*^, *Nes-Met* ^*d/flox*^, *Nes-Met* ^*2P/flox*^, and *Nes-Met* ^*2S/flox*^ neonates (P2; n = 6 for each group of animals) at C8-T1 level. In this region, MN clusters are organized in two groups (dashed lines). The more ventral cluster (red dashed lines) exhibits less MNs in the *Nes-Met* ^*d/flox*^ mutant, in contrast to *Nes-Met* ^*2P/flox*^ and *Nes-Met* ^*2S/flox*^ mutants, which are comparable to controls. **(D)** Quantification of MN numbers throughout the brachial region of *Nes-Met* ^*d/flox*^, *Nes-Met* ^*2P/flox*^, and *Nes-Met* ^*2S/flox*^ P2 mutants, showing percentages of MN loss per section. Positions of the C8, T1, and T2 DRGs are indicated by black lines. In the C8-T1 region, where percentages of MNs loss in *Nes-Met* ^*d/flox*^ mutants are highest, *Nes-Met* ^*2P/flox*^ and *Nes-Met* ^*2S/flox*^ mutants show non-significant MN loss, and are significantly different from *Nes-met* ^*d/flox*^ mutants. Student’s t test: *P < 0.05, **P < 0.01, ***P < 0.001. Error bars represent standard error of the mean.

We next sought to determine whether such flexibility in signalling requirements for HGF/Met-dependent MN survival also occurs *in vivo*. In contrast to early stages, once MNs have become dependent on muscle-derived signals for survival, depletion of limb muscles in *met-*signalling mutants causes limb MN death [16]. This is illustrated by the disappearance of *Pea3*-expressing neurons (Figure 6A) and the drastic reduction in the number of *Ret*-expressing MNs occurring in *met*^*d/d*^ embryos at E14.5 (Figure 6B), as well as by the proportional reductions of *Pea*3-expressing or *Ret*-expressing MNs in the brachial spinal cord of *met*^*2P/2P*^ and *met*^*2S/2S*^, *met*^*2Pneo/2Pneo*^ and *met*^*2Sneo/2Sneo*^ embryos with reduced volumes of muscles (Figure 6, Additional file 1: Figure S1A, and Additional file 4: Figure S4A,D,E). Therefore, the process of MN death that matches the number of MNs with the number of myofibres in the cognate target muscle, occurs between E12.5 and E14.5. This constitutes genetic dating of the onset of muscle dependency, consistent with previous observations [4,16].

We have recently used conditional inactivation of *met* owing to Cre-mediated excision in the nervous system (*Nestin-Cre*) to demonstrate the direct control by HGF/Met of the *in vivo* survival of a MN pool in the C8-T1 region corresponding to the *pectoralis minor* muscle [4]. As complete *Nestin-Cre*-mediated Met ablation occurred only after the onset of NOCD, this strategy allowed us not only to preserve muscle development, hence maintaining an unaltered amount of muscle in the limb, but also to bypass the early function of Met in the specification of the *Pea3* pools and in motor nerve growth (Figure 6C,D) [4]. This genetic paradigm also allowed us to directly assess the net effect on MN survival attributed to Met-signalling changes in MNs *in vivo*. We therefore generated neuronal-specific-switch mutants in which one allele of *met* is floxed by *loxP* sites and the other allele carries the *met* signalling mutation: *Nestin-Cre; met*^*d/flox*^, *Nestin-Cre; met*^*2P/flox*^ and *Nestin-Cre; met*^*2S/flox*^ (referred to as *Nes-Met*^*d/flox*^*, Nes-Met*^*2P/flox*^ and *Nes-Met*^*2S/flox*^, respectively). In contrast to *Nes-Met*^*d/flox*^ mutants, the numbers of MNs measured in the *Nes-Met*^*2P/flox*^ and *Nes-Met*^*2S/flox*^ mutants were equivalent to WT, with only minor non-significant reductions at T1 level (Figure 6C,D, Additional file 6: Figure S6). Thus, survival of the *pectoralis minor* MN pool is not compromised in neural-specificity-switch Met mutants, as was found in our culture assays. Altogether, these results show that signalling routes downstream of the Met RTK can substitute for each other to promote MN survival and that those solicited by Met^2P^ and Met^2S^ are equivalent in their efficacy in accomplishing this task. Such findings illustrate the remarkable degree of plasticity of Met signalling cascades ensuring survival, in contrast to the strict specificity required for Met-dependent axon guidance and to the partial flexibility for *Pea3* identity acquisition by the HGF/Met system.

## Discussion

**Figure 7:**
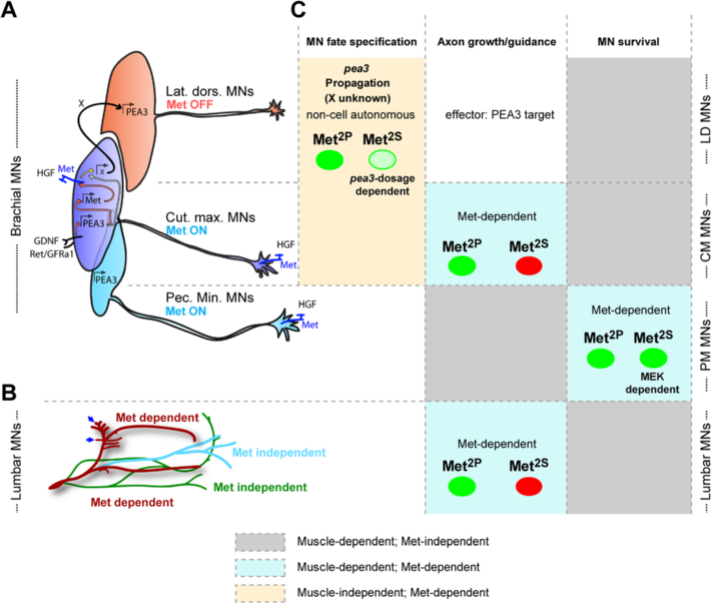
**Summary of the signaling modalities characterizing each of the functions of HGF/Met in MNs. (A,B)** Schematic summary of the MN populations in which HGF exerts its functions, highlighting which subsets express Met. **(A)** Scheme representing the three subsets of *Pea3*-expressing MNs, and the mechanism by which GDNF and HGF cooperate to establish *Pea3* expression domain, based on [16]: GDNF acts on Ret/GFRa1 expressing “pioneer” neurons (dark blue pool, believed to largely match the CM MN pool in general) , to induce *Pea3* expression. *Pea3* in turn is required to induce expression of the HGF receptor Met. HGF acts on the same “pioneer” neurons, once they express Met, to trigger the production by these neurons of a signal (referred to as X) that induces *Pea3* expression in additional neurons (“recruited”). This leads to the lateral enlargement of the CM pool (within which *Met* expression propagates as well), and to the recruitment of more anterior neurons (red, largely matching the LD MN pool), which will not express *Met*. **(B)** Scheme representing lumbar axonal MN projections in the hindlimb, with colours distinguishing Met-dependent from Met-independent guidance. **(C)** Summary table of the various biological aspects of neuromuscular development involving HGF/Met. Columns distinguish the nature of the biological response (cell fate specification, axon growth/guidance, and survival); rows distinguish the MN populations, matching them to their position in the schemes in (**A**, top) and (**B**, bottom). The table indicates for each response/MN population the respective degree of muscle dependency, the corresponding function of HGF/Met, whether it is cell autonomous or not, and the degree of execution by signalling solicited by Met^2P^ and Met^2S^ specificity-switch mutants (with green indicating complete execution, and red indicating abrogation).

Signalling pathways are solicited to convey information from membrane receptors activated by growth factors to coordinate a set of outcomes including short-term transcription-independent responses and long-term gene-expression changes. When the same receptor is employed to trigger distinct biological responses in a given cell type, each response can be achieved through the activation of distinct signalling cascades. The degree to which a given signalling cascade is uniquely required for a specific biological response, or can be substituted by an alternative pathway, depends on the cell type, the nature of the response, the strength of the signal and the robustness of the signalling network available for this biological outcome. We have investigated this issue *in vivo* in the context of several biological requirements of the HGF/Met system in MNs, using a set of mouse lines carrying signalling mutations of Met that modulate the phosphotyrosine binding preferences, and thereby limit the range of signalling effectors mobilised by the modified receptor. We showed that in MNs, alternative HGF-induced Met cascades such as the PI3K or Src/Mek pathways, although biochemically or genetically distinguishable, are interchangeable to ensure MN survival, and MN specification, but not for axon guidance (Figure 7).

### Degree of equivalence of signalling cascades and robustness of a biological response

A growing set of data supports the idea that signalling effectors are in charge of selective biological responses following RTK stimulation. While the view 15 years ago conveyed the idea that a given signalling cascade would be uniquely required for a given response, it has now emerged that several alternative pathways, although distinct from each other by their components, can substitute for one another to activate a given biological response. Therefore, the robustness of a biological outcome correlates with the capacity of cells not only to buffer variations of signal intensities [37], but also to offer a multiplicity of signalling molecules that can efficiently replace each other to ensure a given response [38,39]. Here we asked to which extent several biological responses to one pleiotropic RTK, Met, are compatible with substitutions of signalling route downstream of the receptor for their faithful execution:

- Substitutable pathways, considered permissive for a given biological response, induce a cascade of biochemical events that converge towards and mobilise key activator(s) of this response, compatible with a positive outcome. Considered together, all components of alternative/substitutable pathways capable of mediating a given response can constitute an equivalence group, where the lack of one component (or lowered signalling levels by one component) can easily be compensated for by the network of available relationships between the others [39–41]. This concept has been instrumental in understanding pathways leading to cancer, where mutations in any component of a given equivalence group (also called a core pathway) are mutually exclusive and tend not to co-occur in the same tumour [41,42].
- Conversely, a pathway is considered non-permissive when it is not substitutable with another one, and when activation of an alternative pathway will not lead to the same output (even leading in some cases to the opposite outputs, for example attraction versus repulsion in axon guidance).

Whether certain pathways are equivalent (permissive) or not depends, on one hand, on the cell type (whether it is competent to offer a robust plasticity network to buffer signalling changes) and, on the other hand, on the nature of the biological outcome. Our results distinguish three types of biological outcomes, which are discussed in the following paragraphs:

### Non-equivalence of signalling cascades for axon branching towards muscle targets

We showed that switching Met binding preferences towards Src family kinases precludes axonal branching to target muscles by HGF/Met, while accommodating PI3K recruitment ensures correct peroneal nerve patterning in the hindlimb (Figure 7). These differences of biological competences between Met^2P^ and Met^2S^, with respect to axonal growth and intramuscular branching, are important for two main reasons:

1. They allow unambiguous uncoupling of Met requirements for axonal growth from muscle development, and they also allow distinguishing of a nerve pattern defect from the consequences of an altered MN specification process (unlike for brachial *Pea3* MN pools). The amount of muscle mass present in the limbs due to limited myoblast migration varies from embryo to embryo, although to a similar extent in *met*^*2P/2P*^ and *met*^*2S/2S*^ embryos (Figure 2C,D). Cases with no muscles (where the *TA* muscle is completely missing) result in the absence of nerve branches exiting the main peroneal nerve, even in a small subset of *met*^*2P/2P*^ embryos. This reflects the fact that muscles are necessary for sending a branching signal. However, in the presence of equivalent amounts of muscle mass, such branches are unequivocally detected in *met*^*2P/2P*^ but not in *met*^*2S/2S*^ embryos, where the nerve pattern is indistinguishable from that seen in *met*^*d/d*^ mutants. This indicates that Met signalling is essential for the decision of motor axons to exit the main nerve and enter the muscle, provided that the competent signalling effectors are mobilised. According to these results, the muscle-derived signal may be either HGF itself or a protein necessary for HGF to bind/activate its receptor, such as enzymes involved in HGF precursor cleavage and/or in its extracellular diffusion [43–45].
2. The complete inability of the Met^2S^ receptor to execute an axonal branching decision, which contrasts with its capability to trigger MN survival, indicates that Src family kinase activation is inadequate for axonal branching by HGF/Met (or that Src antagonises the response otherwise mediated through another pathway such as PI3K; see below). These results apparently contrast with other ligand/receptor systems in which Src family kinases have been reported to be required for axon guidance but not for survival of MNs [46]. In this study, interfering with Src signalling in developing chicken spinal cords resulted in axonal path-finding errors at the dorsoventral choice point in the limb [46], a decision cooperatively controlled by ephrinA/EphA and GDNF/Ret signalling pathways [16,47–49]. Similarly, inactivation of the *Drosophila* Src homologue causes aberrant midline crossing indicating that Src is required to inhibit crossing of longitudinal axons [50]. A possible explanation for this apparent discrepancy with our results may be that Src family kinases are involved in non-permissive pathways, where they instruct axons of the behaviour they should adopt (whether they should be attracted or repelled) in response to various signalling cues. Consistently with this hypothesis, Src family kinases are involved in repulsive responses downstream of ephrin reverse signalling [51,52], but mediate attractive turning responses to Netrin [53]. Thus, for the HGF/Met system, Src activation by Met^2S^ may have turned an attractive/positive response into a negative/repulsive one, for example by mobilising a cascade normally involved in a negative response to another signal. In such a context, Src activation would therefore not be dispensable, but rather would antagonise the normal function of Met. In contrast, the PI3K pathway appears as a permissive pathway, which may serve as a switch to activate responsiveness or as a rheostat to modulate the intensity of a unique one-sided response of axonal growth [54]. What determines whether Src family kinases will mediate an attractive or repulsive response may be encoded by intrinsic differences between neuron subtypes, for example through differential expression of receptor complexes or signalling effectors involved in gating the nature of this decision [55–57]. An alternative possibility could be that in the WT context, Src family kinase recruitment by Met occurs only in a MN subtype-specific manner; for example Src may not be solicited in those MNs in which Met regulates guidance (CM, peroneal axons). In this context, modifications of the Met binding preference may result in an ectopic Src family kinase activation, thereby removing the pool-specificity and silencing the response of neurons.

### Two non-equivalent pathways can substitute for each other to control cell fate acquisition

In contrast to the clear incapacity of signalling by the Met^2S^ receptor to execute axon guidance decisions, the cascade of events allowing expansion of the *Pea3* expression domain in the brachial motor columns is efficiently executed by both Met^2P^ and Met^2S^ receptors, the two pathways being, however, differentially sensitive to *Pea3* gene dosage (Figure 7). *Pea3* expression was previously shown to be initially activated by GDNF in a subset of brachial MNs [31]. We have previously added another degree of complexity by showing that this induction was restricted to a pioneer group of Met-expressing neurons, in which Met is required to trigger the production of a signal propagating *Pea3* expression to anterior neurons (Figure 7A, Additional file 3: Figure S3 and [16]). With known inducers of *Pea3,* such as FGF8/3 and Wnt1, not being expressed in the pioneer pool, work is currently underway to identify the signal, induced by HGF/Met in pioneer neurons, that is responsible for expansion of the *Pea3* expression domain, not only laterally, within the Met-expressing CM MN pool, but also to Met-negative anterior MNs. The results of the present study indicate that the signalling cascades mobilised by both Met^2P^ and Met^2S^ receptors equivalently allow triggering of the production by pioneer neurons of this signal mediating expansion of *Pea3* expression to recruited neurons. The sensitivity to lowered *Pea3* levels in the Met^2S^ scenario indicates that the responsible signalling cascade involves Pea3 transcriptional activity. This further implies that this *Pea3*-inducing signal must be a *Pea3* target gene. The possibility that *Pea3* could be involved in its own propagation is consistent with the reduced domain of *Pea3-lacZ* expression described for *Pea3*-null embryos [32] and with the fact that Met expression in the pioneer domain is regulated by Pea3 (Additional file 1: Figure S1) [16]. The differential effectiveness of the signalling cascade activated by the Met^2P^ and Met^2S^ receptors suggests that Pea3 transcriptional activity might be regulated by Src but not by PI3K. Pea3-family transcription factors are typically known to be activated by a Ras-Mek axis downstream of FGF signalling [58–62]. A recent study shows that ERK signalling mediates induction of *Etv4* (*pea3*) and *Etv5* expression in sensory neurons by target-derived neurotrophic factors such as NGF [63]. Our own result from MN survival assays suggest that the pathway mobilised by the Met^2S^ receptor for survival also involved the ERK pathway through Mek activation. It is therefore tempting to speculate the existence of a Src-Mek-Pea3 circuit available downstream of HGF/Met in MNs. Consistent with this hypothesis, Pea3 was shown to be involved downstream of v-src signalling to activate transcription of collagenase 1/MMP1 [64].

Moreover, *Pea3* loss-of-function disrupts Src activation and cell mobility in *Pea3*-null fibroblasts, indicating that Src can also be activated and required downstream of Pea3 [65]. Such a reciprocal relationship between Pea3 and Src could explain why a twofold lowering of *Pea3* expression does not impair propagation of *Pea3* expression in a WT Met signalling context, but becomes critical in the context of a signalling-switch mutant of Met predominantly relying on Src activation. In contrast, the fact that signalling by the Met^2P^ receptor is not sensitive to reduced *Pea3* dosage indicates that production of the signal mediation expansion of *Pea3* expression can also be induced by other transcription factor(s). Thus, the robustness of the cell fate specification function triggered by the Met RTK relies on a complex signalling circuit possibly composed of a combination of distinct transcription factors, the activity of which is modulated qualitatively and/or quantitatively by upstream signalling cascades.

### Equivalence of signalling cascades for neuronal survival

Finally, we found that signalling pathways mobilised by either the Met^2P^ or Met^2S^ receptors were equally capable of substituting for the WT pathways in regulating MN survival. This is supported by primary MN culture experiments and by *in vivo* studies on Met-dependent *pectoralis minor* MN pool survival [4] using neuronal-specificity-switch Met mutants. *In vitro*, survival by HGF in *met*^2P/2P^ MNs is selectively mediated by PI3K, but not by Src or Mek, whereas in *met*^2S/2S^ MNs, survival requires Src and Mek, but not PI3K. Neuronal survival by numerous neurotrophic factors, including HGF in MNs [35], involves the traditional PI3K-Akt pathway. Akt regulates components of the apoptotic machinery, such as Bad, a pro-apoptotic member of the Bcl-2 family, and Forkhead box transcription factors, which are involved in expression of other death genes [66,67]. Besides this main road, alternative pathways can also be employed for cell survival [68]. For example, a number of studies have provided evidence that Src is required for survival promoted by GDNF, by cytokines, such as CNTF, or by the P2Y2 receptor, in cultured embryonic MNs or osteoclasts. Nevertheless, in these cell types Src survival properties are still dependent on the PI3K-Akt pathway [10,35,69–71]. The requirement of Src and Mek, but not PI3K, for MN survival by Met^2S^ implies either a parallel action of Src and Mek-ERKs pathways, or the existence of a circuit involving activation of Mek-ERKs by Src. A previous report on chick MNs has indicated that Mek signalling is dispensable for their survival [36]. However, saturating concentrations of HGF (10 ng/ml) were used in this study, whereas we favoured assessing the signalling required for survival using HGF doses more similar to the physiological context (0.4 ng/ml or 2 ng/ml). It is possible that with saturating amounts of ligand, pathways such as PI3K are over-activated to an extent that is sufficient for MN survival, even in the absent of other signalling components such as Mek. In contrast, with the lower concentration of HGF that we used (closer to the levels occurring *in vivo*), the level of activation of each individual pathway may not be sufficient to promote survival independently of the others. This scenario coincides with the signalling requirements for survival we have evidenced *in vivo*, in the context of our neuronal-restricted Met specificity-switch mutants.

Intriguingly, the signalling pathways initiated by Met^2P^ and Met^2S^ receptors are adequate for survival of MNs, but not of embryonic hepatocytes both *in vitro* and *in vivo* [23–25]. Such a paradox illustrates the extent to which the signalling efficacy for a given biological response is influenced by the cell type. This may be related to intrinsic factors, such as the biological state of cells (e.g. proliferating versus quiescent), or to extrinsic factors, such as extracellular levels of stress signals. Another reason may be differences in expression patterns and levels of signalling components in embryonic MNs versus hepatocytes. In this respect, Additional file 7: Figure S7 offers an illustration of the expression patterns of several members of various signalling families, based on the GenePaint database [72]. It is interesting to note that several adaptors expressed at high levels in the nervous system, some of them even specifically in brachial MN pools, appear either not to be expressed in the liver or to be expressed at much lower levels.

The high plasticity of signalling networks controlling MN survival is particularly intriguing, when taking into account the unexpected discovery that PI3K, Src and Mek-ERK pathways are all necessary downstream of the WT Met receptor, each of them being, however, insufficient on its own to provide a robust survival response in the absence of the other pathways *in vitro*. This contrasts with the fact that Src is dispensable downstream of Met^2P^ and PI3K is dispensable downstream of Met^2S^. A possible explanation of this apparent discrepancy could be that in MNs, Met specificity-switch receptors behave as mild gain-of-function mutants for some pathways, while being incapable of activating the others. This increased level of activation of individual signalling routes downstream of Met^2P^ or Met^2S^ would raise their activity above a threshold necessary to activate effectors of survival, whereas this threshold is only reached by the combined activity of PI3K, Src and MAPK pathways downstream of WT Met. According to this scenario, such increased levels above the threshold could alleviate the simultaneous requirement for the other pathways to induce a survival response successfully in MNs. As mentioned above, this contrasts with hepatocytes, in which signalling initiated by Met^2P^ or Met^2S^ receptors is insufficient to ensure a survival response to HGF [23–25], thus indicating that the gain-of-function character is conditioned by the competence of the cell type. Molecular analysis of signalling circuits downstream of Met WT and Met mutants would offer the possibility of uncovering the components and levels of their activation that ensure the robustness of survival circuits in MNs. However, addressing this question would require detailed molecular and biochemical analyses that are technically not compatible with the number of MNs accessible from embryos, and would be challenged by the switch in biological responses and in MN subtypes occurring throughout development.

## Conclusions

How can we interpret the specificity of signalling pathways necessary for axonal growth compared to the plasticity allowed when executing RTK-induced MN survival or specification responses? Axon guidance is a complex example of a biological response regulated by several non-equivalent signalling components. Their activation leads to choices between multiple qualitatively different outcomes, such as stop, go, turn, exit, etc., thereby requiring intercalation of multiple signalling systems to gate the decision-making process at the level of the growth cone. Such a context implies that some signalling cascades need to be specifically associated with selective outcomes rather than being permissive, for the proper integration of multiple instructive axon guidance signals. In contrast, MN survival and specification are dichotomic choices at the cell level, integration of which at the tissue level involves the quantitative assessment of individual neuron responses to the limited amount of neurotrophic factors produced by target tissues. Adaptation of the MN numbers in each pool to the size of its cognate muscle target is achieved by a mechanism capable of measuring accurately the amount of target-derived signal available, and of adjusting this amount with respect to an intrinsic threshold within each cell, to block the apoptotic cascade or to activate the specification program. Likewise, raising RTK signalling levels above endogenous thresholds can enhance the neuroprotective effect and limit susceptibility to MN diseases [73]. The plasticity allowing several alternative pathways to substitute for each other to achieve such responses confers robustness to the mechanisms coordinating the development and size of the neuromuscular system.

## Methods

### Animals and genotype analysis

The generation of the different signalling alleles of the *met* gene used in this study (*met*^*d*^, *met*^*2P*^ and *met*^*2S*^) has been described previously [9,15]. The original neo^+^ alleles, which contained a neo selection cassette flanked by LoxP sites, were referred to as met^2Pneo^ and met^2Sneo^. Alleles without *neo* were obtained after excision of the *neo* cassette by crossing mutant mice with the Deleter-Cre transgenics [9]. Genotype analysis by PCR was performed as described [9,12]. The *met*^*LacZ*^ allele is an alternative knockout/knock-in allele of *met* in which the *LacZ* reporter gene also reflects expression of the endogenous *met* gene [4]. In the original allele, *LacZ* expression is conditioned by the removal of a Lox-stop-Lox cassette [4]. In the allele used here, by crossing with a deleter-cre line, we have derived a mouse line in which the stop cassette has been permanently deleted. Genotype analysis was performed by PCR as described [4]. *Pea3-lacZ* mice were used with the permission of Arber and Jessell, and genotyped as previously described [32]. Animals were maintained and sacrificed in accordance with institutional guidelines. Adult mice were euthanised by cervical dislocation.

### *In situ* hybridisation

Embryos were collected in phosphate-buffered saline (PBS) and fixed in 4% paraformaldehyde (PFA). ISHs were performed with the relevant RNA probe on either whole-mount embryos, dissected limbs or spinal cords, according to previously published procedures [16]. Whole spinal cord ISH was performed as described [74], with digoxigenin-labelled RNA probes for *met*, *MyoD* (obtained from Ponzetto), *Pea3*, (from Jessell), *Ret* (from Rosenthal) and *ChAT* (choline acetyl transferase; from Henderson). Spinal cords were flat mounted as open-book preparations with MNs on the upper side, and imaged using a Zeiss Axiophot (Marly le Roi, France) or Leica stereomicroscope (Wetzlar, Germany). Whole-mount embryos were partially cleared in 50% glycerol and imaged using a stereomicroscope.

### X-Gal and Salmon-Gal staining for β-galactosidase activity

Staining for β-galactosidase activity was performed using two alternative methods. For spinal cords of embryos carrying the *pea3*^*LacZ*^ allele, we employed the traditional protocol using X-Gal substrate in combination with ferric and ferrous ions. Briefly, spinal cords were dissected from freshly collected embryos, fixed in 4% PFA in PBS (40 min), rinsed in PBS, and incubated in X-Gal in combination with potassium ferricyanide and ferrocyanide (FeCN). To detect β-galactosidase in the spinal cords of embryos carrying the *met*^*LacZ*^ allele, we used an alternative method based on Salmon-Gal (6-chloro-3-indolyl-beta-D-galactopyranoside from Appolo scientific, Maschester, UK) in combination with tetrazolium salt [75]. Briefly, spinal cords were dissected from freshly collected embryos, fixed in 1% PFA and 0.2% glutaraldehyde in PBS (10 min), rinsed three times in PBS, then incubated for a minimum of 7 h overnight at 37°C in pre-stain solution without substrate (potassium ferricyanide 200 mM, potassium ferrocyanide 200 mM, 4 mM MgCl2 and 0.04% NP40 in PBS) to reduce endogenous β-galactosidase activity. Samples were then rinsed three times in PBS, and incubated in staining solution (1× PBS, with 0.04% NP40, 2 mM MgCl_2_, 1 mg/ml Salmon-Gal (stock solution 40 mg/ml in dimethyl sulfoxide (DMSO)) and 0.33 mg/ml NBT (stock solution 75 mg/ml in 70% DMF)), for about 30 to 40 min at 37°C. In all cases (X-Gal and Salmon-Gal), staining was terminated by rinsing in PBS, and post-fixing in 4% PFA. Spinal cords were flat mounted in 1 volume glycerol/1 volume 4% PFA for imaging.

### Embryonic motor neuron cultures

E12.5 mouse embryos were collected in Hibernate (E) medium (Life Technology) with B-27 supplement (Life Technology) and kept on ice until the dissection, while the genotype was determined by PCR. Ventral spinal cords from an equal number of WT and mutant embryos were dissected. MNs were isolated from either brachial + lumbar, or from cervical + thoracic + sacral segments, by a previously described method [34] involving a combination of centrifugation on BSA cushions and a metrizamide density gradient centrifugation. At the end of the procedure, the cell suspension was highly enriched in MNs. For survival assays, MNs were plated in polyornithine/laminine-coated four-well tissue culture dishes (1,500 neurons per well) in Neurobasal medium (Life Technology, Saint Aubain 91190, France). Recombinant neurotrophic factors and inhibitors were added 2 h after seeding. MNs were kept for 3 days either in Neurobasal medium or in the presence of GDNF or HGF (both obtained from R&D, Minneapolis, MN, USA). All inhibitors (LY294002, PD98059, PP2 and PP3) were obtained from Calbiochem (Darmstadt, Germany). PP3 was used as a negative control for PP2 (not shown). MN survival was quantified by counting large bright unipolar neurons with long axonal processes on the whole area of each well.

### Whole-mount anti-neurofilament immunohistochemistry

Embryos were collected in cold PBS, fixed for 2 h in 4% PFA in PBS, and post-fixed overnight in Dent’s fixative (80% methanol, 20% DMSO) at 4°C. Whole-mount anti-neurofilament immunohistochemistry was performed as previously described [29]. Briefly, embryos were bleached for 4 h in 6% H_2_O_2_ in Dent’s solution and further rehydrated through a progressive methanol series. Antibody incubations were performed in 80% newborn calf serum, 20% DMSO, 0.5% triton with thimerosal. Antibodies used were the following: anti-neurofilament antibody (2H3 and DSHB) and HRP-conjugated goat anti-mouse antibody (Sigma). Staining was developed using DAB Tablets (Sigma, Saint-Quentin Fallavier, France). Embryos were cleared for imaging in BABB (1:2, benzyl alcohol/benzyl benzoate).

### Image processing and analyses

Quantification of signal intensity was performed using the ImageJ software. Briefly, images of ISH were first converted to a grey scale and inverted to a negative scale (with the highest signal intensity matching being white and the lowest black). Signal intensity was measured along a horizontal line of a given pixel length (matching half a flat-mounted spinal cord, always positioned where indicated with dotted lines on Figure 3). After background and threshold subtraction, the values were averaged between several samples of each genotype (considering left and right sides separately, for the number of spinal cord sides used, for each plot indicated in the corresponding figures) to generate an average signal distribution plot. The total signal intensity was also calculated for each sample, and plotted individually (Figures 2 and 5, Additional file 1: Figure S1 and Additional file 4: Figure S4).

### Statistical analysis

Results were expressed as mean ± standard error of the mean. Statistically significant differences were assessed by unpaired Student’s *t*-test when data were showing a normal distribution, and by the Mann–Whitney test otherwise. * indicates *P* < 0.05 and ** indicates *P* < 0.001.

## Additional files

Additional file 1: Figure S1. Figure S1. Met-deficiency-triggered reduced limb muscle volume does not cause MN loss at E12.5. **(A)** Analysis of muscle migration by whole mount *MyoD* ISH on E11.5 (top row) or E12.5 (bottom row) embryos, showing forelimbs Of WT and *met* signalling mutants (n = 3 embryos per genotype and stage). **(B)** *Ret* ISH in brachial spinal cords from E12.5 WT, *met* ^*d/d*^, *met* ^*2P/2P*^, *met* ^*2S/2S*^ embryos. **(C)** The size of the Ret-expressing brachial MN pools was assessed using measurements with Image J of *Ret* ISH staining intensity along the orange dotted line at C7 level. Each plot represents the average signal distribution of the indicated number of spinal cord sides (*met* ^*+/+*^: n=18; *met* ^*d/d*^: n=14; *met* ^*2P/2P*^: n=10; and *met* ^*2S/2S*^: n=8). **(D)** Met expression was followed by Salmon-Gal staining in spinal cords from E12.5 *met* ^*LacZ/+*^; *met* ^*LacZ/d*^; *met* ^*LacZ/2P*^; and *met* ^*LacZ/2P*^ embryos. Images show the brachial region of the same spinal cords shown in Figure 3C. **(E)** Quantification of the size of the *Met*-expressing brachial neurons along the orange line at C7 level. Each plot represents the average signal distribution of the indicated number of spinal cord sides (*met* ^*LacZ/+*^: n=22; *met* ^*LacZ/d*^: n=8; *met* ^*LacZ/2P*^: n=18; and *met* ^*LacZ/2P*^: n=10). **(F, G)** Quantifications and statistical analyses of the sum of signal intensity corresponding to measurements of Ret ISH staining **(F)**, or *Met* ^*LacZ*^ expression **(G)**, based on intensity plots in **(C)** or **(E)**, respectively. Numbers of samples are as indicated in **(C)** and **(E)**. At E12.5, the size of the Ret-expressing MN population is not significantly altered in *met* mutant embryos, as MN numbers are independent of muscle mass at this stage. In contrast, analysis of *met* ^*LacZ*^ expression confirms that *met* is required for its own expression in the C7-C8 brachial pool, signalling via either PI3K or Src being equally efficient to ensure establishment of *met* expression domain. Additional file 2: Figure S2. Altered peroneal nerve guidance in absence of Met signalling cannot be attributed to lowered *Pea3* expression. **(A)** Scheme of the lumbar spinal nerves and plexuses at hindlimb levels (same as in Figure 3A) based on a lateral view of an E11.5 WT embryo, showing that the sacral plexus is located caudal to the sciatic plexus, and collects axons mostly originating from L6 and S1 spinal segments. **(B)** Double ISH with *Met* (blue) and *Pea3* (red) probes (left), or with the *Pea3* probe only (right), was performed for E12.5 WT spinal cords to show their respective expression patterns in the lumbar region in apposition with spinal segments and the corresponding plexuses shown in **(A)**. Overall a longitudinal column expressing *Pea3* lies more ventral (closer to the midline) than the Met-expressing motor neurons at L4–5–6 levels, while the caudal part of the *Pea3* domain maps to the sacral segments, thus corresponding to projections to the sacral plexus. Therefore, *Pea3* is not expressed in *Met*-expressing peroneal motor neurons (yellow dotted pools). **(C)** Analysis of *Pea3* and its target gene *Sema3E* expression in lumbar spinal cords of WT and *met* ^*d/d*^ embryos confirms that, as previously shown for the brachial region, *Met* is also required for expansion of expression of *Pea3* and *Sema3E* in the lumbosacral region (*n* = 4 spinal cords for each probe and each genotype). **(D)** Anti-neurofilament (NF) immunohistochemistry for hindlimbs of WT and *Pea3* ^*LacZ/LacZ*^ E12.5 embryos, showing that *Pea3* functions are dispensable for peroneal nerve guidance. **(E)** High magnification of the area from a WT E13.5 hindlimb stained with anti-NF antibody, where side branches exit the deep peroneal nerve to make contacts with muscles. The right panel shows the same picture a white line highlighting the nerve, and green lines highlighting side branches. Additional file 3: Figure S3. Scheme representing the three subsets of *Pea3*-expressing MNs, and the mechanism by which GDNF and HGF cooperate to establish *Pea3* expression domain, based on [16]: Step1: prior to GDNF secretion by the plexus mesenchyme, Pea3 is not expressed, but a subset of neurons express the receptor Ret/GFRa1 coupe. Step2: GDNF acts on Ret/GFRa1 expressing “pioneer” neurons (dark blue pool, believed to largely match the CM MN pool), to induce *Pea3* expression. *Pea3* in turn is required to induce expression of the HGF receptor Met. Step3: HGF acts on the same “pioneer” neurons, once they express Met, to trigger the production by these neurons of a signal (referred to as X) that induces *Pea3* expression in additional neurons (“recruited”). This leads to the lateral enlargement of the CM pool (within which *Met* expression propagates as well), and to the recruitment of more anterior neurons (red, largely matching the LD MN pool), which will not express *Met*. Expression of Pea3 in the Pec Min MN pool appears to occur independent of GDNF [31], but which peripheral factor is required for this induction has not been identified. Additional file 4: Figure S4. MN survival is proportional to the amount of muscle in developing limbs in embryos with attenuated expression levels of Met-signalling variants. **(A)** Analysis of muscle migration by whole-mount *MyoD ISH* in WT, *met* ^*2Pn/2Pn*^ and *met* ^*2Sn/2Sn*^ E12.5 embryos at forelimb levels (*n* = 3 embryos for each genotype). Note that the presence of a neo cassette in the *met* locus further reduces the ability of mutant receptors to promote myoblast migration and colonisation of the limb, due to attenuation of receptor expression levels. Consequently, muscle masses in *met* ^*2Pn/2Pn*^ and *met* ^*2Sn/2Sn*^ embryos are reduced compared to *met* ^*2P/2P*^ and *met* ^*2S/2S*^ embryos (see Additional file 1: Figure S1A,B). **(B–E)** ISH was performed with *Pea3* **(B,D)** and *Ret* **(C,E)** RNA probes on spinal cords from E12.5 **(B,C)** and E14.5 **(D,E)** WT, *met* ^*2Pn/2Pn*^ and *met* ^*2Sn/2Sn*^ embryos (with a minimum *n* = 3 spinal cords for each probe, stage and genotype). Quantification of the lateral expansion of the *Pea3* domain **(B)** and assessment of MN numbers through *Ret* ISH **(C)**, were performed by measuring signal intensity; each plot represents the average signal distribution measured on the indicated number of spinal cord sides (WT *n* = 14 for *Pea3 and n* = 18 for *Ret*; *met* ^*2Pn/2Pn*^ *n* = 6 for each probe; *met* ^*2Sn/2Sn*^ *n* = 6 for each probe), the two sides being considered separately, along the red dotted line for each image. Measurements were performed with ImageJ, after negative conversion and background subtraction. This analysis shows that prior to muscle dependency, overall MN numbers are unaffected by reduced muscle mass and that the Met^2P^ and Met^2S^ receptors, in spite of reduced *met* expression levels in *met* ^*2Pn/2Pn*^ and *met* ^*2Sn/2Sn*^ embryos, retain an intermediate but comparable capacity to promote expansion of the *Pea3* expression domain (as quantified in **(C)**, *P* > 0.89). **(D,E)** After the onset of muscle dependency (at E14.5), as expected, the reduced MN survival in *met* ^*2Pn/2Pn*^ and *met* ^*2Sn/2Sn*^ embryos correlates with reduced muscle masses. Again, *met* ^*2Pn/2Pn*^ and *met* ^*2Sn/2Sn*^ embryos do not appear different from each other. Additional file 5: Figure S5. Detection of Met-expressing MNs in the brachial spinal cord of Met signalling mutants. Met expression was detected in E13.0 spinal cords from WT, *met* ^*d/d*^, *met* ^*2P/2P*^ and *met* ^*2S/2S*^ embryos (minimum *n* = 3 spinal cords per genotype) by ISH with an RNA probe matching the *met* extracellular domain, which was unchanged by the genetic modifications to the *met* locus. The spinal cords (unopened) are imaged from their left side, at the brachial level, with anterior to the left and posterior to the right. Additional file 6: Figure S6. MN numbers in neural-specific Met signalling mutants. MN numbers were quantified at P2 in the brachial region (C8-T1) of *Nes-Met* ^*2P/flox*^ (*n* = 8) **(A)** and *Nes-Met* ^*2S/flox*^ (*n* = 7) **(B)** mutants compared to controls (*met* ^*flox/+*^ *n* = 4). *ChAT*-expressing MNs were quantified on cryosections shown in Figure 5C. Positions of the C8, T1 and T2 DRGs are indicated by black lines. These graphs correspond to the graphs shown in Figure 5D displaying percentage of loss. Additional file 7: Figure S7. Potential for plasticity of signalling pathways illustrated through expression patterns of signalling effectors. Sagittal sections of E14.5 mouse embryos hybridised with the indicated probe (expression data are extracted from the GenePaint database). Brachial MN pools are indicated by arrows. **(A)** Src family kinases and associated effectors. **(B)** PI3K subunits and downstream effectors (Akt1). **(C)** MAPK family and effectors. When the indicated gene is expressed in brachial MN pools, a magnification of the area is shown.

## Abbreviations

BSA: Bovine Serum Albumine
CM: Cutaneous maximus
cut.pn: cutaneous (sensory) peroneal
d.pn: deep peroneal
dm: distal muscle mass (un-split dorsal hand muscles)
DMF: Dimethylformamide
DMSO: Dimethyl-sulfoxide
DRG: Dorsal Root Ganglion
EDL: Extensor digitorum longus
edl: *extensor digitorum longus*
edl.b: branches entering the *extensor digitorum longus* muscle
Etv4: Ets variant gene 4 (formerly called Pea3 for polyomavirus enhancer activator 3)
GDNF: Glial-derived Neurotrophic Factor
HGF: Hepatocyte Growth Factor
ISH: *in situ* hybridization
LD: Latissimus dorsi
LMC: Lateral Motor Column
MN: Motor Neuron
Muscles: ta: *tibialis anterior*
NBT: Nitro Blue Tetrazolium
neo: Neomycin resistance gene
Nerves: co.pn: common peroneal
PBS: Phosphate-buffered saline
PCR: Polymerase chain reaction
pd: *peroneus digiti* (includes *peroneus digiti quarti* and *peroneus digiti quinti*)
PFA: Paraformaldehyde
PI3K: Phosphatidylinositol 3-kinase
pl: *peroneus longus*
PTB: Phosphotyrosine Binding Domain
s.pn: superficial peroneal
SH2: Src homology domain type 2
SH3: Src homology domain type 3
TA: Tibialis anterior
ta.b: branches entering the *tibialis anterior* muscle
WT: Wild-type
