# Supplementary figures and images for: Plasticity versus specificity in RTK signalling modalities for distinct biological outcomes in motor neurons
Source: BMC Biol. 2014 Aug 14;12:56. doi: 10.1186/s12915-014-0056-6 (PMC4169644; doi:10.1186/s12915-014-0056-6)

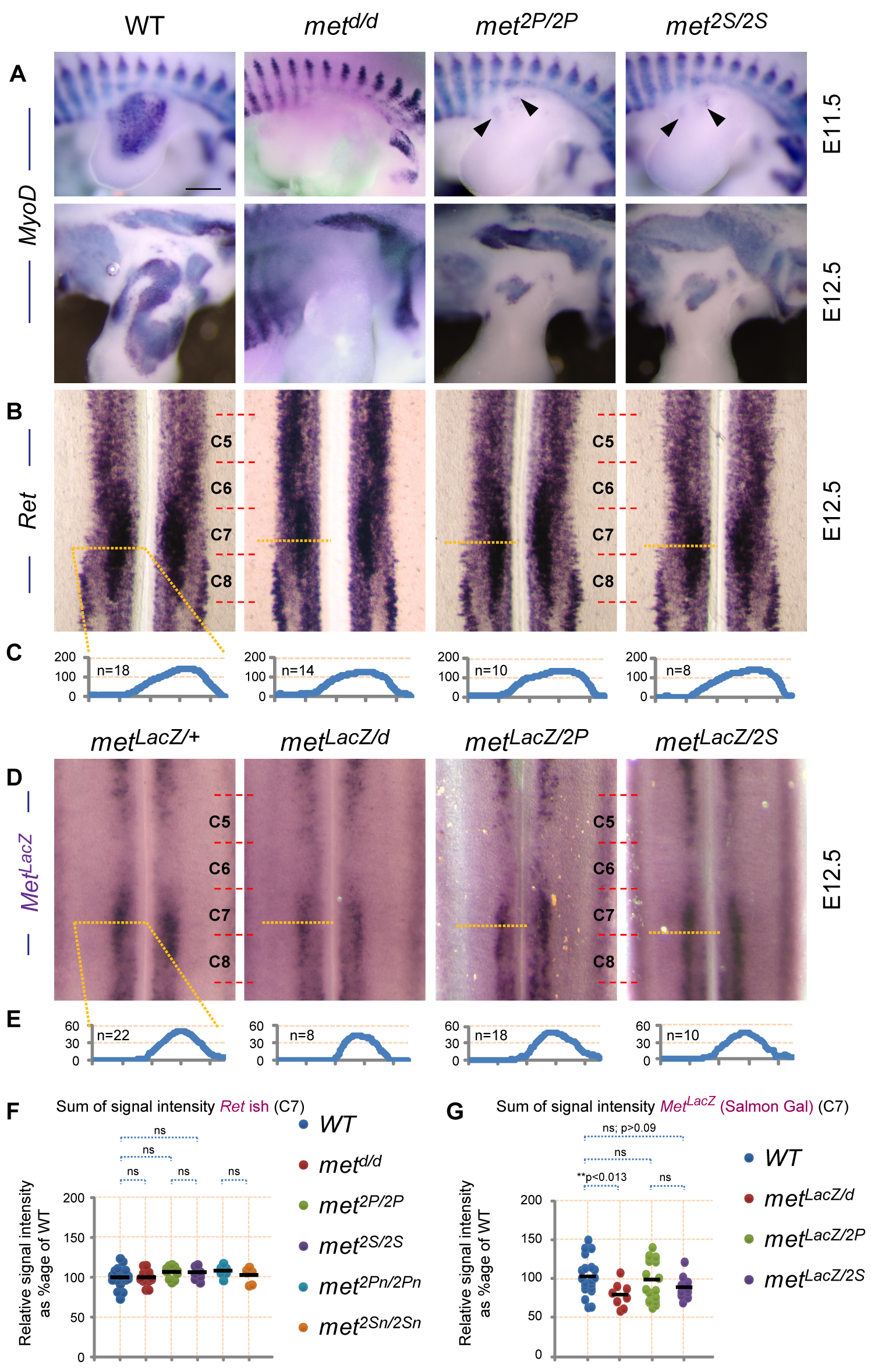

Supplement: Additional file 1: Figure S1. — Figure S1. Met-deficiency-triggered reduced limb muscle volume does not cause MN loss at E12.5. (A) Analysis of muscle migration by whole mount MyoD ISH on E11.5 (top row) or E12.5 (bottom row) embryos, showing forelimbs Of WT and met signalling mutants (n = 3 embryos per genotype and stage). (B) Ret ISH in brachial spinal cords from E12.5 WT, met d/d, met 2P/2P, met 2S/2S embryos. (C) The size of the Ret-expressing brachial MN pools was assessed using measurements with Image J of Ret ISH staining intensity along the orange dotted line at C7 level. Each plot represents the average signal distribution of the indicated number of spinal cord sides (met +/+: n=18; met d/d: n=14; met 2P/2P: n=10; and met 2S/2S: n=8). (D) Met expression was followed by Salmon-Gal staining in spinal cords from E12.5 met LacZ/+; met LacZ/d; met LacZ/2P; and met LacZ/2P embryos. Images show the brachial region of the same spinal cords shown in Figure 3C. (E) Quantification of the size of the Met-expressing brachial neurons along the orange line at C7 level. Each plot represents the average signal distribution of the indicated number of spinal cord sides (met LacZ/+: n=22; met LacZ/d: n=8; met LacZ/2P: n=18; and met LacZ/2P: n=10). (F, G) Quantifications and statistical analyses of the sum of signal intensity corresponding to measurements of Ret ISH staining (F), or Met LacZ expression (G), based on intensity plots in (C) or (E), respectively. Numbers of samples are as indicated in (C) and (E). At E12.5, the size of the Ret-expressing MN population is not significantly altered in met mutant embryos, as MN numbers are independent of muscle mass at this stage. In contrast, analysis of met LacZ expression confirms that met is required for its own expression in the C7-C8 brachial pool, signalling via either PI3K or Src being equally efficient to ensure establishment of met expression domain. [file 12915_2014_56_MOESM1_ESM.tif]

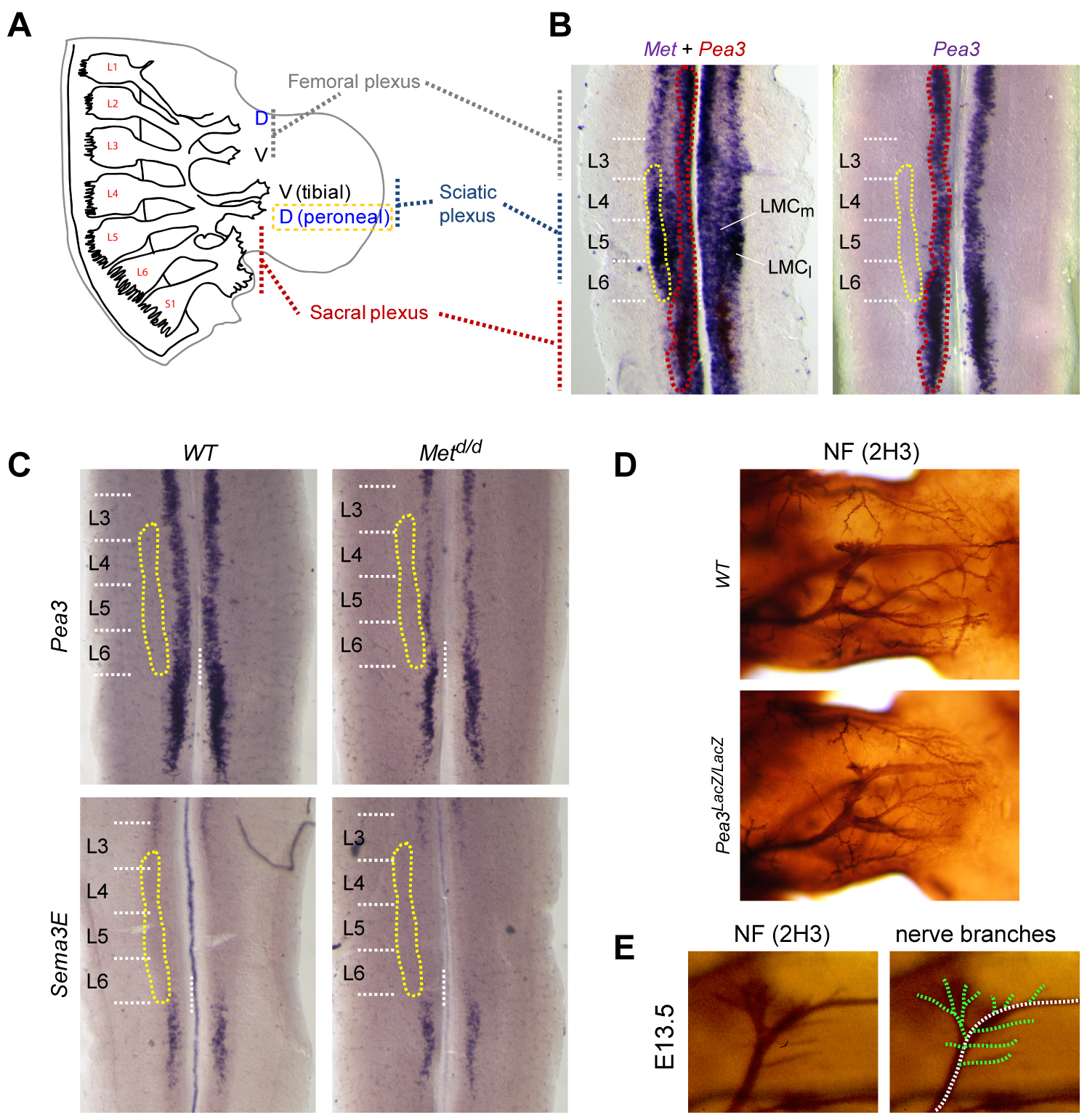

Supplement: Additional file 2: Figure S2. — Altered peroneal nerve guidance in absence of Met signalling cannot be attributed to lowered Pea3 expression. (A) Scheme of the lumbar spinal nerves and plexuses at hindlimb levels (same as in Figure 3A) based on a lateral view of an E11.5 WT embryo, showing that the sacral plexus is located caudal to the sciatic plexus, and collects axons mostly originating from L6 and S1 spinal segments. (B) Double ISH with Met (blue) and Pea3 (red) probes (left), or with the Pea3 probe only (right), was performed for E12.5 WT spinal cords to show their respective expression patterns in the lumbar region in apposition with spinal segments and the corresponding plexuses shown in (A). Overall a longitudinal column expressing Pea3 lies more ventral (closer to the midline) than the Met-expressing motor neurons at L4–5–6 levels, while the caudal part of the Pea3 domain maps to the sacral segments, thus corresponding to projections to the sacral plexus. Therefore, Pea3 is not expressed in Met-expressing peroneal motor neurons (yellow dotted pools). (C) Analysis of Pea3 and its target gene Sema3E expression in lumbar spinal cords of WT and met d/d embryos confirms that, as previously shown for the brachial region, Met is also required for expansion of expression of Pea3 and Sema3E in the lumbosacral region (n = 4 spinal cords for each probe and each genotype). (D) Anti-neurofilament (NF) immunohistochemistry for hindlimbs of WT and Pea3 LacZ/LacZ E12.5 embryos, showing that Pea3 functions are dispensable for peroneal nerve guidance. (E) High magnification of the area from a WT E13.5 hindlimb stained with anti-NF antibody, where side branches exit the deep peroneal nerve to make contacts with muscles. The right panel shows the same picture a white line highlighting the nerve, and green lines highlighting side branches. [file 12915_2014_56_MOESM2_ESM.tif]

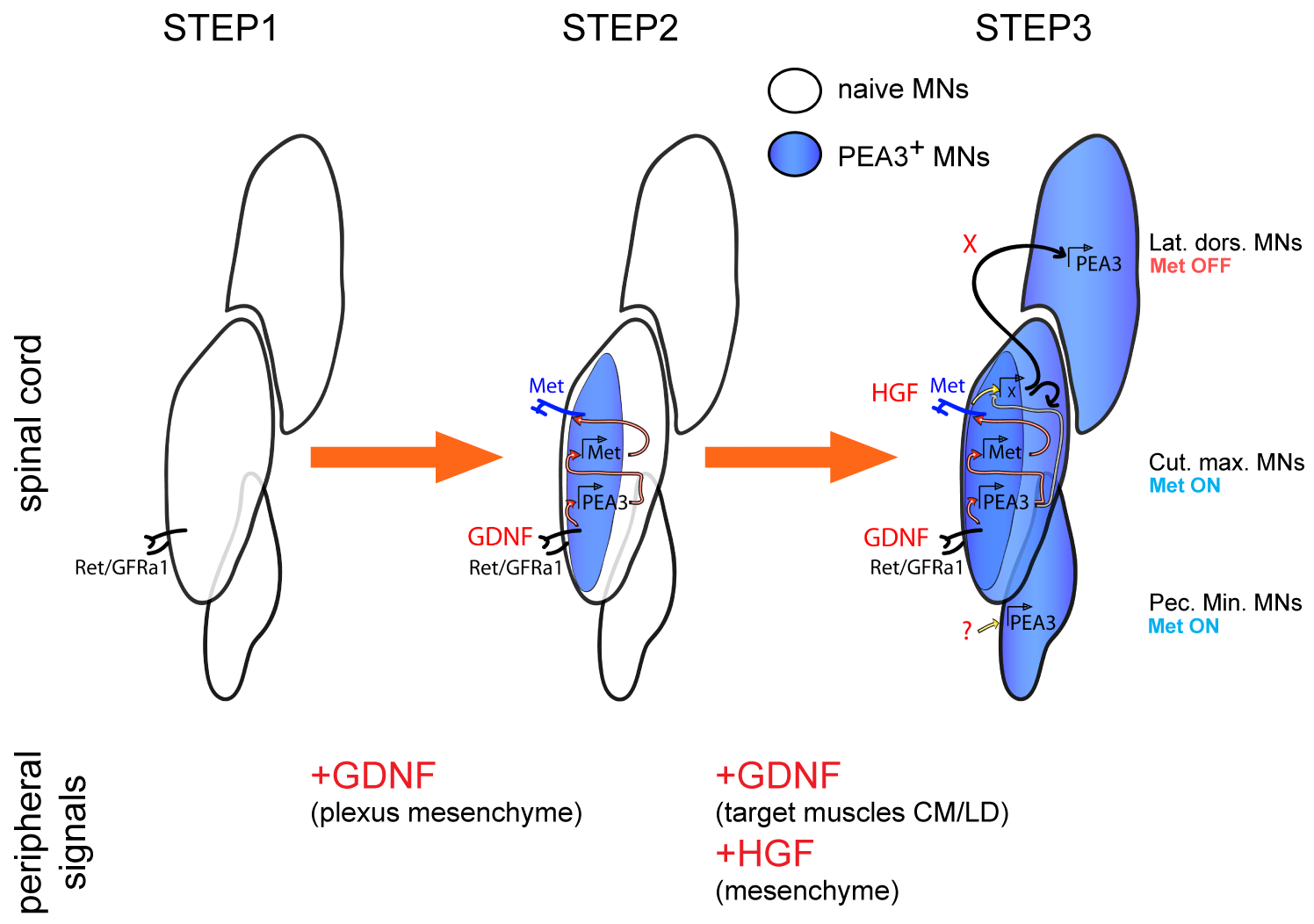

Supplement: Additional file 3: Figure S3. — Scheme representing the three subsets of Pea3-expressing MNs, and the mechanism by which GDNF and HGF cooperate to establish Pea3 expression domain, based on [16]: Step1: prior to GDNF secretion by the plexus mesenchyme, Pea3 is not expressed, but a subset of neurons express the receptor Ret/GFRa1 coupe. Step2: GDNF acts on Ret/GFRa1 expressing “pioneer” neurons (dark blue pool, believed to largely match the CM MN pool), to induce Pea3 expression. Pea3 in turn is required to induce expression of the HGF receptor Met. Step3: HGF acts on the same “pioneer” neurons, once they express Met, to trigger the production by these neurons of a signal (referred to as X) that induces Pea3 expression in additional neurons (“recruited”). This leads to the lateral enlargement of the CM pool (within which Met expression propagates as well), and to the recruitment of more anterior neurons (red, largely matching the LD MN pool), which will not express Met. Expression of Pea3 in the Pec Min MN pool appears to occur independent of GDNF [31], but which peripheral factor is required for this induction has not been identified. [file 12915_2014_56_MOESM3_ESM.tif]

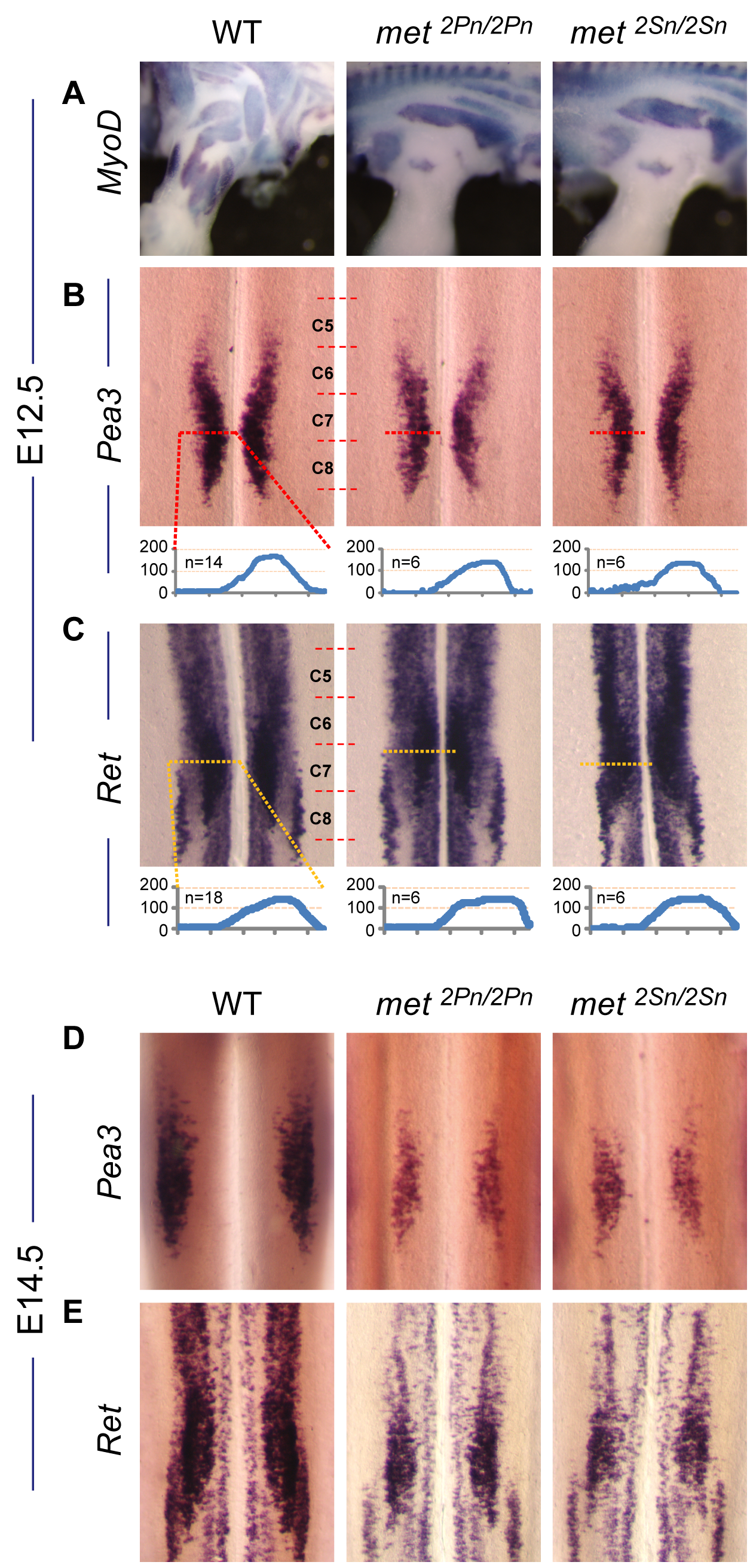

Supplement: Additional file 4: Figure S4. — MN survival is proportional to the amount of muscle in developing limbs in embryos with attenuated expression levels of Met-signalling variants. (A) Analysis of muscle migration by whole-mount MyoD ISH in WT, met 2Pn/2Pn and met 2Sn/2Sn E12.5 embryos at forelimb levels (n = 3 embryos for each genotype). Note that the presence of a neo cassette in the met locus further reduces the ability of mutant receptors to promote myoblast migration and colonisation of the limb, due to attenuation of receptor expression levels. Consequently, muscle masses in met 2Pn/2Pn and met 2Sn/2Sn embryos are reduced compared to met 2P/2P and met 2S/2S embryos (see Additional file 1: Figure S1A,B). (B–E) ISH was performed with Pea3 (B,D) and Ret (C,E) RNA probes on spinal cords from E12.5 (B,C) and E14.5 (D,E) WT, met 2Pn/2Pn and met 2Sn/2Sn embryos (with a minimum n = 3 spinal cords for each probe, stage and genotype). Quantification of the lateral expansion of the Pea3 domain (B) and assessment of MN numbers through Ret ISH (C), were performed by measuring signal intensity; each plot represents the average signal distribution measured on the indicated number of spinal cord sides (WT n = 14 for Pea3 and n = 18 for Ret; met 2Pn/2Pn n = 6 for each probe; met 2Sn/2Sn n = 6 for each probe), the two sides being considered separately, along the red dotted line for each image. Measurements were performed with ImageJ, after negative conversion and background subtraction. This analysis shows that prior to muscle dependency, overall MN numbers are unaffected by reduced muscle mass and that the Met2P and Met2S receptors, in spite of reduced met expression levels in met 2Pn/2Pn and met 2Sn/2Sn embryos, retain an intermediate but comparable capacity to promote expansion of the Pea3 expression domain (as quantified in (C), P > 0.89). (D,E) After the onset of muscle dependency (at E14.5), as expected, the reduced MN survival in met 2Pn/2Pn and met 2Sn/2Sn embryos correlates with reduced muscle masses. Ag [file 12915_2014_56_MOESM4_ESM.tif]

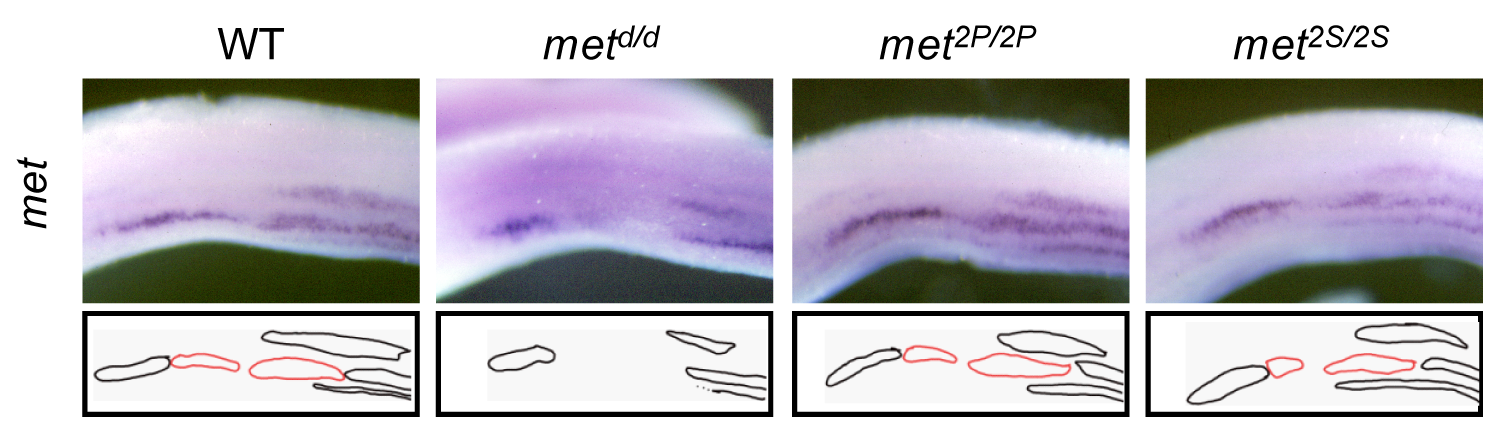

Supplement: Additional file 5: Figure S5. — Detection of Met-expressing MNs in the brachial spinal cord of Met signalling mutants. Met expression was detected in E13.0 spinal cords from WT, met d/d, met 2P/2P and met 2S/2S embryos (minimum n = 3 spinal cords per genotype) by ISH with an RNA probe matching the met extracellular domain, which was unchanged by the genetic modifications to the met locus. The spinal cords (unopened) are imaged from their left side, at the brachial level, with anterior to the left and posterior to the right. [file 12915_2014_56_MOESM5_ESM.tif]

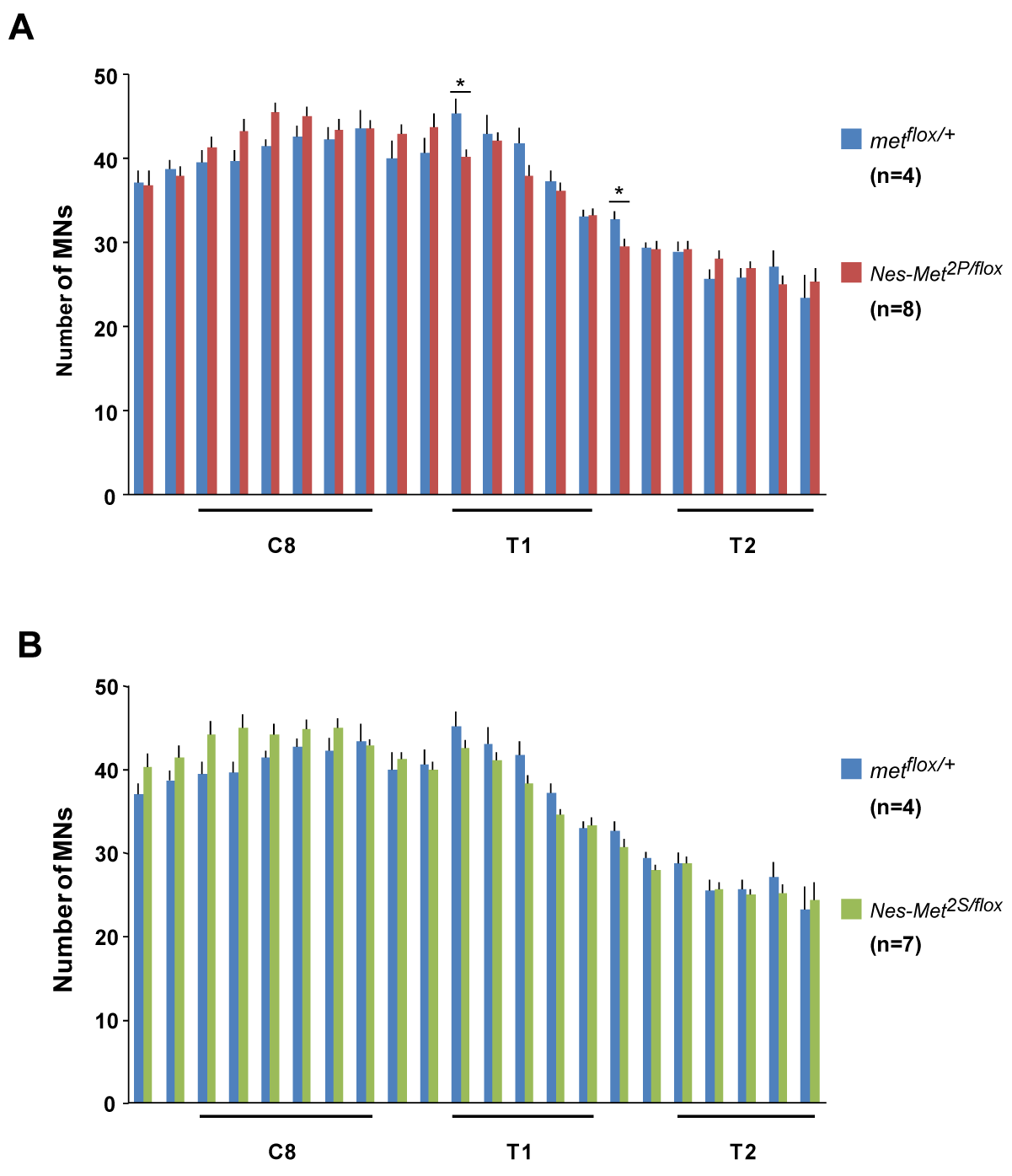

Supplement: Additional file 6: Figure S6. — MN numbers in neural-specific Met signalling mutants. MN numbers were quantified at P2 in the brachial region (C8-T1) of Nes-Met 2P/flox (n = 8) (A) and Nes-Met 2S/flox (n = 7) (B) mutants compared to controls (met flox/+ n = 4). ChAT-expressing MNs were quantified on cryosections shown in Figure 5C. Positions of the C8, T1 and T2 DRGs are indicated by black lines. These graphs correspond to the graphs shown in Figure 5D displaying percentage of loss. [file 12915_2014_56_MOESM6_ESM.tif]

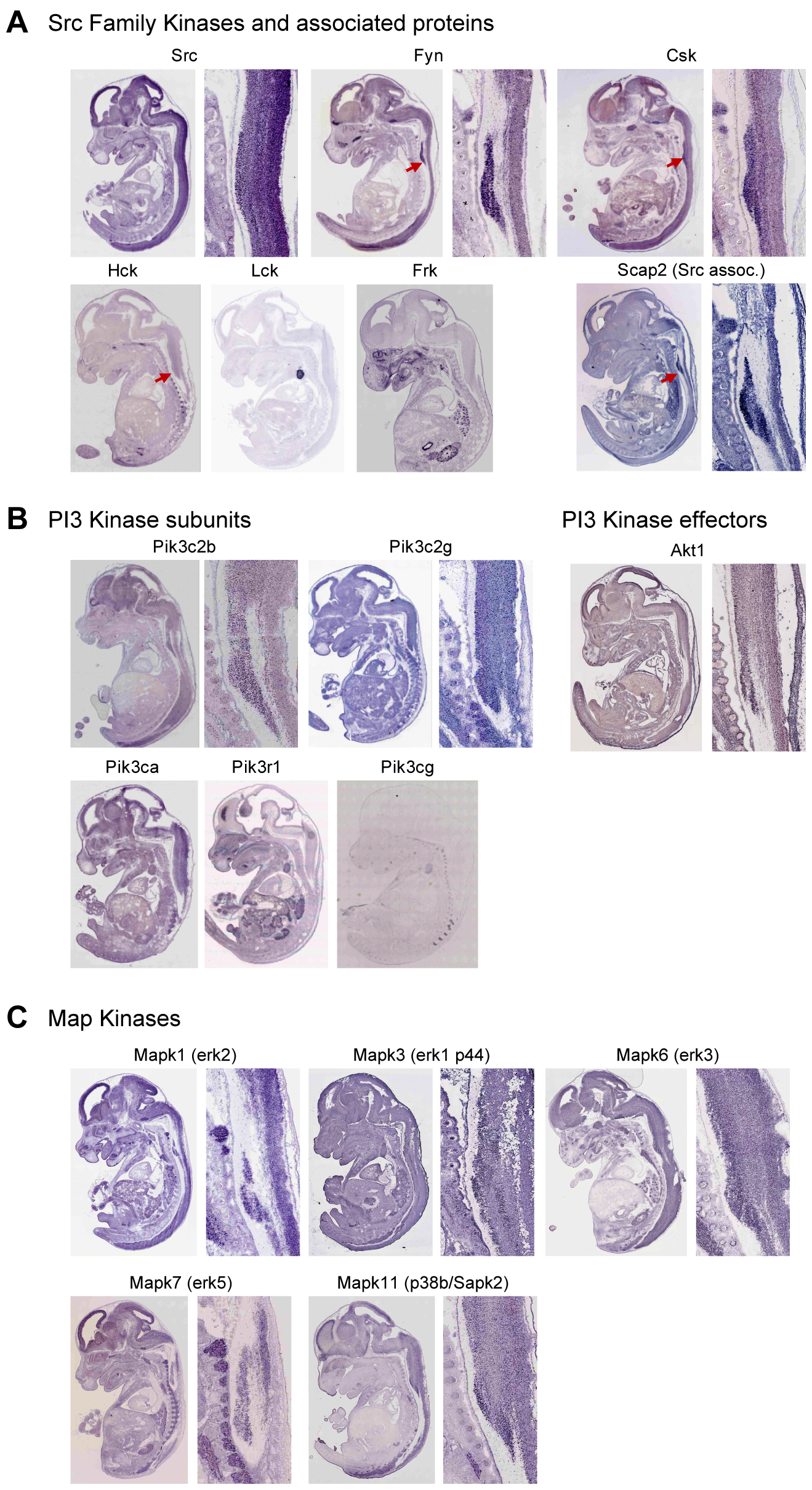

Supplement: Additional file 7: Figure S7. — Potential for plasticity of signalling pathways illustrated through expression patterns of signalling effectors. Sagittal sections of E14.5 mouse embryos hybridised with the indicated probe (expression data are extracted from the GenePaint database). Brachial MN pools are indicated by arrows. (A) Src family kinases and associated effectors. (B) PI3K subunits and downstream effectors (Akt1). (C) MAPK family and effectors. When the indicated gene is expressed in brachial MN pools, a magnification of the area is shown. [file 12915_2014_56_MOESM7_ESM.tif]
